# A phylogenetic analysis of the Primnoidae (Anthozoa: Octocorallia: Calcaxonia) with analyses of character evolution and a key to the genera and subgenera

**DOI:** 10.1186/s12862-018-1182-5

**Published:** 2018-05-02

**Authors:** Stephen D. Cairns, Herman H. Wirshing

**Affiliations:** Department of Invertebrate Zoology, National Museum of Natural History, Smithsonian Institution, Smithsonian Institution, PO Box 37012, MRC 163, Washington, DC, 20013-7012 USA

**Keywords:** Octocoral, Alcyonarian, Phylogenetics, Systematics, Primnoidae, Ancestral state reconstruction

## Abstract

**Background:**

Previous phylogenetic analyses of primnoid octocorals utilizing morphological or molecular data have each recovered evolutionary relationships among genera that are largely incongruent with each other, with some exceptions. In an effort to reconcile molecular-based phylogenies with morphological characters, phylogenetic reconstructions were performed with 33 of 43 primnoid genera using four loci (*mtMutS, COI*, *28S and 18S*), and ancestral state reconstructions were performed using 9 taxonomically relevant characters. In addition, an updated illustrated key to the current 48 genus-level (43 genera, 5 subgenera) primnoids is presented.

**Results:**

Ancestral state reconstruction recovered the ancestral colony shape of primnoids as dichotomous planar. Convergence was detected among all 9 characters, and reversals to the character state of the common ancestor occurred in 4 characters. However, some characters were found to be informative. For example, the weak ascus scale of *Metafannyella* is not likely homologous to the ascus scales of *Onogorgia* and *Fannyella*, and the monophyly of two subgenera within *Thouarella*, which contain polyps in either whorls or an isolated arrangement, was supported. Phylogenetic analyses were generally consistent with previous studies, and resulted in the synonymy of one genus and a subgenus, the elevation of two subgenera, and the transfer of two species back to an original genus. For example, body wall ornamentation of *Fanellia* was re-evaluated, indicating a synonymy with *Callogorgia*; the utility of polyp arrangement for the subgenus *Plumarella (Dicholaphis*) was not supported, and is synonymized with the nominate subgenus *Plumarella (Plumarella*); the subgenera *Plumarella* (*Faxiella*) and *Plumarell*a (*Verticillata*) are raised to generic status; and the two *Plumarella* species (*P. diadema* and *P. undulata*) are transferred back to *Thouarella* based on the homology of their marginal scales.

**Conclusions:**

Altogether, and similar to other octocorallian groups, these results indicate that many of the morphological characters examined among primnoids, particularly colony morphology, are labile and exhibit complex evolutionary histories. However, some morphological characters such as coordination of polyps, presence of the ascus body wall scale, number of rows of body wall scales, and number of marginal scales help identify many clades, and are suitable for robust systematic assessments among primnoids.

**Electronic supplementary material:**

The online version of this article (10.1186/s12862-018-1182-5) contains supplementary material, which is available to authorized users.

## Background

The family Primnoidae, one of 50 families in the subclass Octocorallia [1], ranks fourth in number of species (279 species) and first in generic diversity (43 genera + five additional subgenera) within the subclass Octocorallia [2–4]. It has been called the “quintessential deep-water octocorallian family” ([2]: page 1) based on its geographic ubiquity (found from the Arctic to Antarctic) and its propensity to live in deep water. Two hundred sixty-nine of its 279 species (96%) have bathymetric ranges extending below 100 m, most living far deeper, as much as 6400 m. Indeed, of the 39 octocoral species known to exist deeper than 3000 m, 22 (56%) of them are primnoids ([5]: Table 1). Some primnoids, e.g., *Primnoa*, are large (up to 2 m tall and 7 m in width) and locally abundant, forming refugia for fish [6] and other invertebrates [7, 8], especially on seamounts [9]. In addition, because of the solid, layered axis and long life spans of many primnoid species, isotopic analyses of their axes have been used to determine paleotemperatures [10].

**Table 1:** List of primnoid genera and subgenera, with junior synonyms

***Abyssoprimnoa*** **Cairns, 2015**	
[*Acanthoprimnoa* Cairns & Bayer, 2004]	
***Aglaeoprimnoa*** **Bayer, 1996**	
*Ainigmaptilon* Bayer, 1980	
*Armadillogorgia* Bayer, 1980	
*Arntzia* López-González, Gili & Orejas, 2002	
[*Arthrogorgia* Kukenthal, 1908]	
***Australogorgia*** **Cairns & Bayer, 2009**	
*Callogorgia* Gray, 1858 (=*Xiphocella* Gray, 1870; *Fanellia* Gray, 1870)	
*Callozostron* Wright, 1885	
***Calyptrophora*** **Gray, 1866**	
***Candidella*** **Bayer, 1954 (=** ***Stenella*** **Gray, junior homonym)**	
*Convexella* Bayer, 1996	
*Dasystenella* Versluys, 1906 (=*Tauroprimnoa* Zapata-Guardiola & López-González, 2010)	
*Digitogorgia* Zapata-Guardiola & López-González, 2010	
*Fannyella (Fannyella*) Gray, 1872 (=*Ascolepis* Thomson & Rennet, 1931)	
*F. (Cyathogorgia*) Cairns & Bayer, 2009	
***F. (Scyphogorgia*) Cairns & Bayer, 2009**	
***Faxiella*** **Zapata-Guardiola & López-González, 2012**	
[*Helicoprimnoa* Cairns, 2012]	
*Heptaprimnoa* Cairns, 2012	
***Loboprimnoa*** **Cairns, 2016**	
*Metafannyella* Cairns & Bayer, 2009	
[*Metanarella* Cairns, 2012]	
[*Microprimnoa* Bayer & Stefani, 1989]	
*Mirostenella* Bayer, 1988	
*Narella* Gray, 1870 (=*Stachyodes* Wright & Studer in Studer, 1887)	
[*Narelloides* Cairns, 2012]	
*Onogorgia* Cairns & Bayer, 2009	
*Ophidiogorgia* Bayer, 1980	
***Pachyprimnoa*** **Cairns, 2016**	
*Paracalyptrophora* Kinoshita, 1908	
***Paranarella*** **Cairns, 2007**	
*Parastenella* Versluys, 1906	
*Perissogorgia* Bayer & Stefani, 1989	
*Plumarella (Plumarella*) Gray, 1870 (=*Dicholaphis* Kinoshita, 1907)	
*Primnoa* Lamouroux, 1812 (=*Lithoprimnoa* Grube, 1861)	
[*Primnocapsa* Zapata-Guardiola & López-González, 2012]	
*Primnoeides* Studer, 1887	
*Primnoella* Gray, 1858	
[*Pseudoplumarella* Kűkenthal, 1915]	
[*Pterostenella* Versluys, 1906]	
***Pyrogorgia*** **Cairns & Bayer, 2009**	
[*Scopaegorgia* Zapata-Guardiola & López-González, 2010]	
*Thouarella (Thouarella*) Gray, 1870 (=*Amphilaphis* Studer & Wright in Studer, 1887; =*Rhopalonema* Roule, 1908; =*Primnodendron* Nutting, 1912; =*Parathouarella* Kükenthal, 1915; =*Epithouarella* Kűkenthal, 1915; =Group 1 sensu Taylor & Rogers, 2009)	
*T. (Euthouarella*) Kükenthal, 1915 (=?*Diplocalyptra* Kinoshita, 1908; =Group 2 sensu Taylor & Rogers, 2009)	
*Tokoprymno* Bayer, 1996	
***Verticillata*** **Zapata-Guardiola, López-González & Gili, 2012**	

Text in boldface indicate taxa added in the study; Unaltered text indicate genera reported by Taylor & Rogers [3]; Text in brackets indicate genera yet to be sequenced

Primnoids have been heavily studied over the last 150 years, leading Kükenthal [11] to state as early as 1915 that they may be the most thoroughly investigated family in the order Gorgonacea (now Alcyonacea). At that time of intense nationalistic efforts of deep-sea dredging, the authors Kinoshita, Versluys, Nutting, and Kükenthal himself (see [2] for historical resume of the family) laid the foundation of primnoid taxonomy. This tradition was continued by Frederick M. Bayer, considered the authority of octocoral taxonomy of the latter half of the twentieth century (see [12]), who published 27 papers (25% of all his papers on octocorals) exclusively or primarily on primnoids, in which he described 67 new species and 14 new genera. A student of Bayer, Cairns, also published 26 papers to date (76% of his octocoral papers) on primnoid taxa. Cairns & Bayer [2] published a revision of the primnoid genera, accompanied by a key to the genera, a list of all taxa (at that time 233 species and 36 genera), and a morphology-based phylogenetic analysis. Subsequently, Zapata-Guardiola & López-González published seven papers on Antarctic primnoids between 2009 and 2012, Taylor et al. [13] produced a monograph of the speciose genus *Thouarella*, and Taylor & Rogers [3] provided a list of all described primnoid taxa at that time (266 species and 41 genera). In addition, Cairns [14] added to the primnoid fauna of the Aleutian Islands, New Zealand [15, 16], and the Clarion-Clipperton Fracture Zone [5].

Many of the morphological characters traditionally used to delineate suborders, families, and genera among octocorals generally do not correspond well with genetically defined evolutionary lineages [3, 17–21]. Mapped morphological characters and ancestral state reconstructions over octocoral phylogenies of various groups indicate that many morphological characters used for taxonomic delineations are likely extensively homoplasious (i.e., resulting from convergent evolution) [22–24], but may also consist of single origins [24]. For example, molecular-based studies have shown branching morphology to be a highly labile character among genera of the Isididae [23] and the Plexauridae [22], as well as the Primnoidae in a morphology-based analysis [2]. However, the evolution of branching morphology in ellisellid octocorals demonstrate a largely irreversible directionality that suggests genetic constraints to character reversal [25].

In primnoids, molecular approaches have examined population/species-level interactions including mechanisms of species diversification among select primnoid taxa [26, 27], and phylogenetic reconstructions have revealed Primnoidae to be reliably monophyletic [3, 18, 28] with the Chrysogorgiidae as a sister group to the family [3, 29]. Taylor & Rogers [3] generated a primnoid phylogeny with representatives of 24 genera (with 2 subgenera) and 64 species. Many clades were well-supported, and the deeper clades (i.e., backbone) of the tree were assigned to four principal clades that largely corresponded to a sub-Antarctic and non sub-Antarctic division. Within each of the four principal clades, some genera were recovered as monophyletic (*Narella*, *Parastenella*, *Paracalyptrophora*, *Dasystenella*, *Primnoeides*, *Mirostenella*, and *Fannyella*), and in other cases they were poly- or paraphyletic (*Callogorgia*, *Fanellia*, *Primnoella*, *Plumarella*, and *Thouarella*). Subsequently, Taylor & Rogers [30] provided a phylogenetic analysis based on 29 primnoid genera where they synonymized *Digitogorgia* with *Primnoeides* and implied a possible synonymy of *Narella*, *Parastenella*, and *Primnoa*. In each study, Taylor & Rogers [3, 30], the significance of the recovered tree topologies with respect to morphological characters, and their evolution, were discussed in several taxa. However, a robust analysis of morphology and character evolution within the greater primnoid phylogeny was not a primary focus of either study. In order to better understand the biological diversity among primnoid genera, and how to identify them, with an emphasis on morphology and character evolution, 33 of the 43 currently described primnoid genera [9 additional genera (and 3 subgenera) plus the 24 publically available genera from Taylor & Rogers [3]; sequences from Taylor & Rogers [30] were not available prior to analyses for this study] were sequenced for two mitochondrial (*mtMutS* and *COI*) and two nuclear rDNA loci (*28S*, *18S*). Ancestral state reconstructions were performed on nine taxonomically important characters, and the implications of the mapped morphological characters, with respect to the recovered phylogeny, were reviewed. In addition, a diagnostic key to aid in the identification of primnoid genera is provided.

## Methods

### Sample collection

Primnoid colonies were sampled from the collections of the Smithsonian National Museum of Natural History (NMNH). Collection dates for each specimen varied from 1965 to 2014, and in combination were collected virtually circumglobally (Additional file 1: Table S1A). Of the 43 currently described primnoid genera and 5 additional subgenera (Table 1), 24 genera and 2 additional subgenera from Taylor & Rogers [3] were used (Table 1, unaltered names; Additional file 1: Table S1B). Although attempts were made to sequence all remaining supraspecific primnoid taxa, based on museum specimens, sequence data was obtained for 9 additional genera (plus 3 subgenera) and 34 new species (53 total specimens) (Table 1, boldface; Additional file 1: Table S1A, with Genbank accession numbers). The degradation of tissue quality in older NMNH specimens (*see also* [31]) likely did not allow for the successful processing of the remaining 10 genera for molecular analyses (Table 1, in brackets). The final dataset contained 178 taxa (33 genera and 78 species) and 1 outgroup (Additional file 1: Table S1A and B).

### DNA extraction, amplification, and sequencing

For DNA extraction, three to four whole polyps were sampled from ethanol-preserved (70% - 95%) or dried colonies. Samples that were stored in ethanol and collected more recently generally performed better in downstream analyses. However, some colonies > 50 years in storage yielded suitable DNA for single locus Sanger sequencing (Additional file 1: Table S1A). Total genomic DNA was isolated using either a proteinase K/phenol extraction method as implemented by AutoGenprep965 (AutoGen, Inc., Holliston, MA), or with DNeasy® blood and tissue kits (Qiagen, Inc., Valencia, CA, USA). Final elution was in 80μl of each respective manufacturer-provided buffer. The polymerase chain reaction (PCR) was used to amplify two mitochondrial loci (*mtMutS* and *COI*) and two nuclear (*28S* and *18S*) loci. Forward priming for *mtMut*S was performed by either ND42599F: 5′- GCCATTATGGTTAACTATTAC-3′ [32] or AnthoCorMSH: 5′- AGG AGA ATT ATT CTA AGT ATG G -3′ [33]. The reverse primer was Mut3458R: 5′ – TSG AGC AAA AGC CAC TCC -3′ [32]. For *COI*, COI-LA-8398-F: 5′ – GGA ATG GCG GGG ACA GCT TCG AGT ATG TTA ATA CGG - 3′, and COIoct-R: 5′ – ATC ATA GCA TAG ACC ATA CC – 3′ [34] were used. For nuclear locus *28S*, Far: 5′ – CAC GAG ACC GAT AGC GAA CAA GTA – 3′, and either Rab: 5′ – TCG CTA CGA GCT TCC ACC AGT GTT T - 3′ or Rar: 5′ – TCA TTT CGA CCC TAA GAC CTC -3′ [35] were used. For *18S*, 18S-A: 5′ – AAC CTG GTT GAT CCT GCC AGT – 3′ [36] and 18S-783R: 5′ – GCC TGC TTT GAA CAC TCT AA TT – 3′ (this study) were used.

PCR amplifications were performed in 10 μL reactions with 1 μL of unquantified genomic DNA and final concentrations of 3 pmol of each primer, 500 μM dNTPS, 3 mM MgCl, 0.25 mg/μL (0.25 μL of 10 mg/μL stock) bovine serum albumin (BSA), and 0.05 U/μL of Biolase™ DNA polymerase (Bioline, Inc) with manufacturer provided buffers. Thermal cycler parameters included an initial denaturing step of 94 °C for 3 min, followed by 35 or 40 rounds of 94 °C for 30 s, 50 °C for 30 or 60 s, 74 °C for 60 s, and a final extension step of 72 °C for 5 min. ExoSAP-IT® (Affymetrix), diluted to a 1:3 concentration of enzyme to purified water and run using a heat profile of 37 °C for 30 min followed by 80 °C for 20 min, was used to neutralize the PCR products. Cycle sequencing was performed with BigDye® Terminater v3.1 (Applied Biosystems). Sephadex™ G-50 Fine (GE Healthcare) was used to purify the cycle-sequenced products, and DNA sequencing was performed on an ABI 3730 at the Laboratories of Analytical Biology (LAB) at NMNH. Sequence contigs were assembled and edited using Geneious Pro 10.0.9 (Biomatters).

### Phylogenetic analyses and ancestral state reconstruction

The phylogenetic trees generated in this study are discussed from the point of view of providing reconciliation to the recovered morphology-based tree and character state transformations from Cairns & Bayer [2]. The higher-level clade numbering system of Taylor & Rogers [3] was maintained to facilitate comparisons, and further augmented with novel clade assignments. Only specimens that yielded two or more sequences of the four targeted loci were used for phylogenetic analyses (sensu [3]), and a concatenated alignment was used for phylogenetic analyses as Taylor & Rogers [3] previously demonstrated that a combined molecular dataset yielded the best results for primnoids. The final dataset contained 178 ingroup taxa (33 genera and 78 species) and one outgroup taxon, the chrysogorgiid *Radicipes stonei* Cordeiro, Cairns & Perez 2017 (USNM1418007) (Additional file 1: Table S1A and B). This outgroup was chosen because the family Chrysogorgiidae has been shown to be sister to the Primnoidae [3, 29], and, consequently, a single individual was sufficient to reliably root the primnoid phylogeny.

Each mitochondrial and rDNA locus was aligned using MAFFT v7.309 [37, 38]. The final alignment was 2831 bp – *mtMutS*: 828 bp, *COI*: 675 bp, *28S*:600bp, and *18S*: 728 bp (Additional file 2). Gblocks v0.91b [39], utilized through the *Phylogeny.fr* sever [40], was used to remove ambiguous regions of each *18S* and *28S* alignment. Gblocks options were set to allow less stringent flanking regions and gap positions, and the resulting parameters were - minimum number of sequences for a conserved position: 78; minimum number of sequences for a flanking position: 131 (*28S*), 132 (*18S*); maximum number of contiguous non-conserved positions: 8; minimum length of a block: 10; and allowed gap positions: with half. For *28S*, 78% (600) of the original 767 positions were used. For *18S*, 96% (728) of the original 752 positions were used. The alignment was divided into data blocks separating each gene, and the protein coding genes, *mMut*S and *COI*, were each assigned codon positions. Partition Finder v1.1.0 [41] was used to determine the best partition scheme from those compatible with the program MrBayes 3.1.2 [42] using both AIC and BIC criterions (Additional file 3 Table S2). Preliminary analyses with partition schemes from either AIC or BIC yielded the same tree topology with very similar posterior probabilities (most within +/− 0.05) (data not shown). Analyses using BIC are presented here.

Phylogenetic reconstruction was performed using Bayesian inference (BI) performed with MrBayes 3.1.2 [42], and maximum likelihood (ML) with RAxML v8 [43]. All analyses were run on the Smithsonian Institution High Performance Cluster (SI/HPC). Bayesian inference analyses consisted of two independent runs with four chains, with trees sampled every 1000 generations. Markov chain Monte Carlo (MCMC) runs were carried out for 10 million generations. The dataset was partitioned into character sets for each locus, and the partition scheme chosen by Partition Finder was applied (Additional file 3: Table S2). The model parameters statefreq, revmat, shape, pinvar, and tratio were each set to either “link” or “unlinked” based on the chosen partition scheme subsets, and the rate prior (prset ratepr) was set to ‘variable’ and applied to ‘all’. Convergence was determined when the potential scale reduction factor (PSRF) was 1.00 and the average standard deviation of split frequencies was < 0.01. Tracer v1.5 [44] was used to verify that an adequate number of trees were sampled from the posterior distribution (effective sample sizes (ESS) were between 1300 and 8500 for each run), and to confirm the stationarity of the runs. The default ‘burn-in’ of 25% sufficiently removed trees before convergence was achieved. Maximum likelihood with RAxML was run using rapid bootstrap analysis and search for best-scoring ML tree (−f a), the GTRGAMMA model (−m), and one thousand bootstrap replicates.

Consensus trees for each BI and ML analysis (i.e., trees with nodes containing support values > 50 for each method) were the same with the exception of two subclades within a larger well-supported clade of *Thouarella*. Since the BI support values for these clades were relatively higher, but still low (0.70 and 0.72 for BI as opposed to < 50 for ML), for these two subclades, the phylogeny generated from the BI analysis was used to map and reconstruct ancestral character states of nine taxonomically important characters sensu [2] (Table 2) using Mesquite v3.2 [45]. Character states were assigned to taxa at the genus and subgenus levels (Table 2), and the resulting matrix (Additional file 4: Table S3) was used to reconstruct the ancestral states of each character on each node of the phylogeny using maximum likelihood with the MK1 model (Additional file 5: Table S4; Additional file 6: Figure S1a-i) and parsimony methods (Additional file 6: Figure S1a-i).

**Table 2:** Morphological character states and additional subgeneric species-groups delineated for character reconstruction. Numbers in paraenthethes correspond to character designations from Cairns and Bayer [2]

Character 1 (1): Colony Shape	
0, unbranched; 1, dichotomous planar; 2, dichotomous (lyriform); 3, dichotomous (bushy); 4, dichotomous (sparse); 5, sympodial (not used in matrix); 6, opposite pinnate; 7, alternate pinnate; 8, bottlebrush; 9, branching from basal bolus	
Character 2 (5): Coordination of polyps	
0, isolated, without order; 1, spirals (not used in matrix); 2, biserial; 3, paired; 4, in whorls (verticillata); 5, unifacial clusters	
Character 3 (8): Operculum	
0, absent; 1, present	
Character 4 (10): Correspondence of opercular and marginal scales	
0, correspond; 1, no correspondence; 2, regular offset	
Character 5 (15): Ascus Scales	
0, none; 1, present	
Character 6 (16): Number of longitudinal rows of body wall scales	
0, not arranged in rows in adult; 1, eight; 2, seven; 3, six; 4, five; 5, three (not in matrix); 6, two; 7, one; 8, none	
Character 7 (19): Number of scales in each abaxial body wall row or abaxial face	
0, variable, but usually over 5; 1, fixed (3 or 4); 2, fixed (5); 3, fixed (2); 4, none	
Character 8 (22): Infrabasal scales	
0, absent; 1, present (paired); 2, present (unpaired)	
Character 9 (11): Number of marginal scales	
0, seven; 1, eight; 2, more than 8; 3, six; 4, five; 5, four; 6, two	
Subgeneric species-groups:	
*Calyptrophora* (group a): species with a lyrate colony: *C. inornata, C. wyvillei*	
*Calyptrophora* (group b): species with dichotomous branching: *C. microdentata*	
*Narella* (group a): species with planar dichotomous branching: *N. mosaica, N. clavata*	
*Narella* (group b): species with branching from basal bolus: *N. hypsocalyx*	
*Narella* (group c): species with sparse dichotomous branching: *N. macrocalyx, N. arbuscula*	
*Thouarella* (group a): species with isolated polyps and bottlebrush branching: *N. variabilis, T. pendulina, T. chilensis, T. antarctica, T. diadema, T. undulata, T. viridis, T, crenelata*	
*Thouarella* (group b): species with isolated polyps and pinnate branching: *T. brucei*	
*Thouarella* (group c): species with whorled polyps and pinnate branching: *T. laxa*	
*Thouraella* (group d): species with whorled polyps and dichotomous branching: *T. coronata*	

## Results and discussion

### Phylogenetic reconstruction and its reconciliation to morphology

**Fig. 1:**
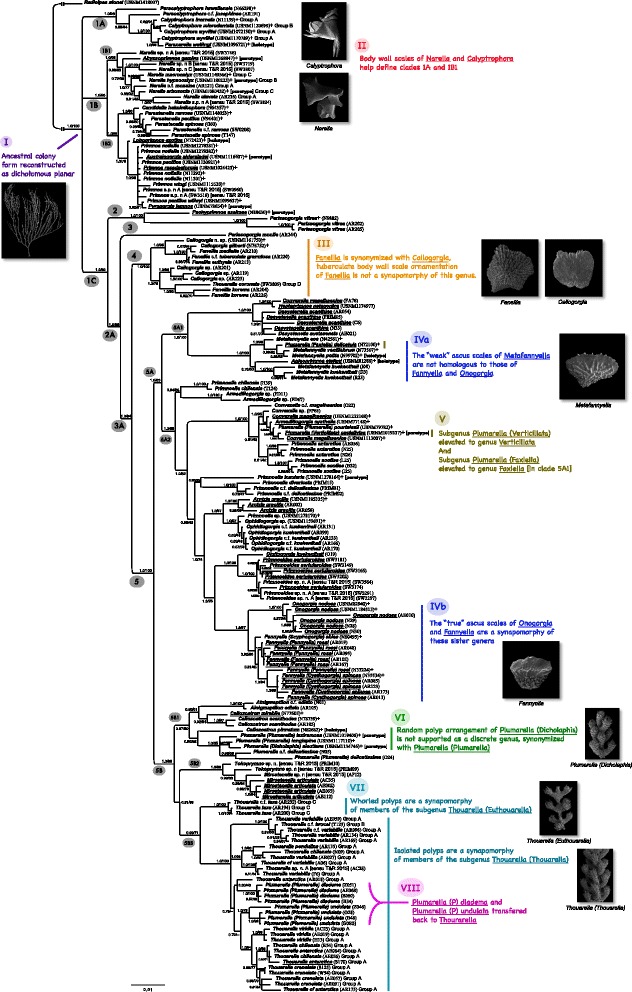
Bayesian phylogram inferred from a four-gene concatenated alignment (*mtMutS*, *COI*, *18S*, and *28S*) with 5 partitions (see Additional file 3: Table S2). Node support contains posterior probabilities on the left, and maximum likelihood (RAxML) bootstrap values on the right. Clades with support values < 50 with both Bayesian inference and maximum likelihood were collapsed. “-” indicates nodes not supported above 50, or not following the branching topology for that method. Type species are underlined, newly sequenced specimens for this study are marked with a “+” after the sample ID, and any holotypes or paratypes sequenced are also labeled as such. Roman numeral inserts highlight the principal findings of the phylogenetic analysis and summarize any taxonomic rearrangements made

The recovered phylogenetic tree (Fig. 1) was largely similar to the tree recovered by Taylor & Rogers [3], and, for consistency, their clade designations 1-5 (see Fig. 1, [3]) were maintained, and novel subclades within them were added. However, two differences in the principal topology were found. Firstly, in Taylor & Rogers [3], members of our clade 5A2 were sister to members of our clade 5B (BI = 0.97 ML = 53, [3], Fig. 1). Here, our clade 5A2 was sister to our clade 5A1 to form the clade 5A (BI = 1.0, ML = 83). Secondly, we did not find sufficient support to keep the two primary subclades within their clade 1 as sister clades (posterior probabilities and bootstrap values were < 50). As a result, this clade was collapsed into two separate clades (clade 1A and 1B, Fig. 1).

#### Clades 1A and 1B

Two of the genera in clade 1A, *Calyptrophora* (Fig. 4f) and *Paracalyptrophora* (Fig. 4h) can be characterized by having some form of dichotomous branching, only two marginal scales (and thus operculars not corresponding to the marginal scales), two rows of body wall scales, only two scales per abaxial body wall row, and infrabasal scales, a morphoclade recognized by Cairns & Bayer [2]. However, the inclusion of *Paranarella* in this clade, a genus Cairns [46] originally thought to be most closely related to *Narella* (a member of clade 1B herein) is puzzling as it differs from *Calyptrophora* and *Paracalyptrophora* in having five marginal scales, eight rows of body wall scales, five scales per abaxial row, and no infrabasal scales (Fig. 4m). More individuals should be analyzed to better assess the placement of this genus. The two branching patterns of *Calyptrophora*, designated as groups a and b (Table 2), do not seem to have any taxonomic significance.

Although no molecular data are available for the genus *Metanarella*, when it was described it was compared to *Narella* and *Paranarella*. This relationship is reinforced by the key to the genera of the Primnoidae (see section *Key to the genera and subgenera of the Primnoidae*), which suggests a placement of this taxon in clade 1A or 1B1. Likewise, although there are no molecular data for the genus *Arthrogorgia*, it is morphologically similar to *Paracalyptrophora* and *Calyptrophora.* Therefore, it is hypothesized to group with these taxa in clade 1A. This placement is supported by the provided key to the primnoid genera, and the morphological cladogram of Cairns & Bayer [2].

**Fig. 2:**
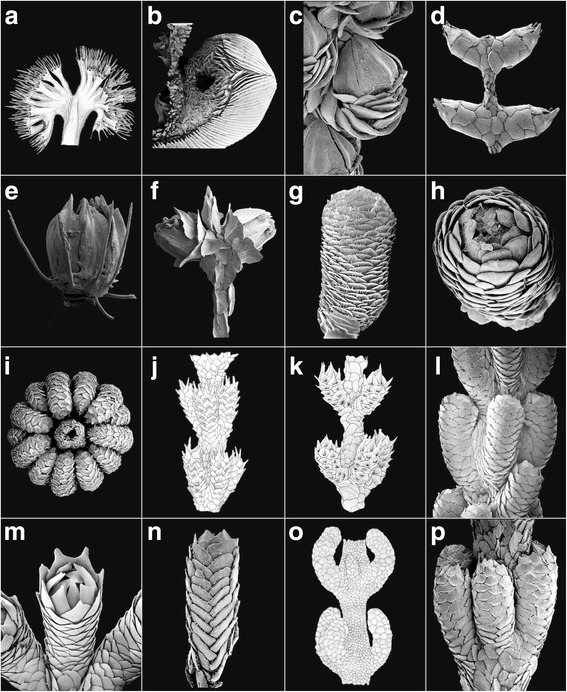
**a** *Ainigmaptilon edisto*, USNM 4950 (holotype); **b** *Armadillogorgia cyathella*, USNM 58166 (holotype); **c** *Pseudoplumarella thetis,* AM G12137 (syntype); **d** *Microprimnoa diabathra*, USNM 79977 (paratype); **e** *Loboprimnoa exotica*, USNM 1278358 (holotype); **f** *Abyssoprimnoa gemina*, USNM 1268847 (holotype); **g** *Aglaoprimnoa stefanii*, USNM 81289 (holotype); **h** *Primnoeides sertularoides*, BM 89.5.27.62 (holotype); **i** *Onogorgia nodosa*, USNM 82945; **j** *Fannyella (Scyphogorgia) abies*, USNM 82981; **k** *Fannyella (Cyathogorgia) spinosa*, USNM 58152; **l** *Metafannyella lepidota*, USNM 84044; **m** *Convexella magelhaenica*, USNM 85306; **n** *Primnoella australasiae*, BM 1983.3.2.13 (syntype); **o** *Pyrogorgia lemnos*, USNM 58392 (holotype); **p** *Fannyella (F.) rossi*, AM G13237 (neotype)

Clade 1B1 consists primarily of members of the well-defined genus *Narella*, but also contains the monotypic *Abyssoprimnoa*, suggested by Cairns [5] to be most similar to *Candidella*, herein classified in the sister-clade 1B2. *Narella* is morphologically similar to the taxa in clade 1A in containing some form of dichotomous branching, and only two rows of large body wall scales, but differs in having three or four scales per abaxial body wall row and four marginal scales (the two abaxial being much larger than the two adaxial) (Fig. 4k). *Abyssoprimnoa* differs from *Narella* in having paired polyps, no body wall scales (only four marginals, unique in the family), and by lacking infrabasal scales (Fig. 2f). Cairns & Bayer [2] similarly placed *Narella* as a possible sister-group to a clade containing the 1A taxa *Calyptrophora* and *Paracalyptrophora*. The various branching patterns of *Narella*, described as groups a-c herein (Table 2), do not seem to have any taxonomic significance. Although no molecular data are available for the genus *Narelloides*, its great similarity to the genus *Narella* (see [16]) suggests a placement in subclade 1B1.

**Fig. 3:**
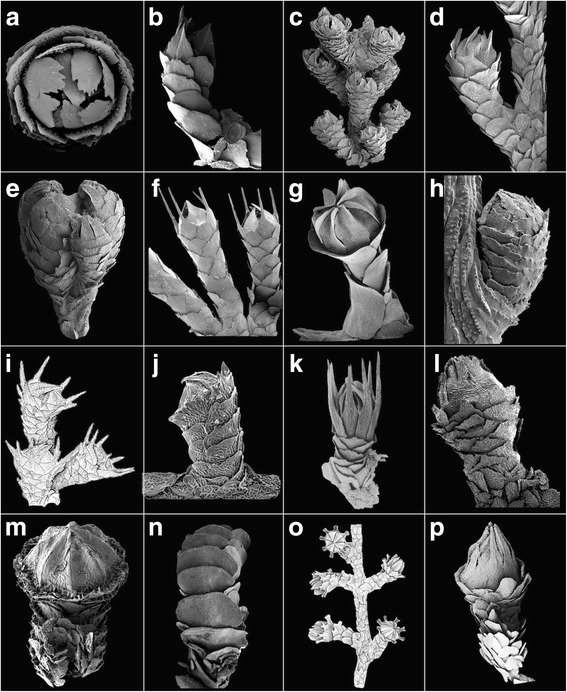
**a** *Digitogorgia kuekenthali*, USNM 1128575; **b** *Scopaegorgia liouvillei*, MNHN OCT 0233 (holotype); **c** *Thouarella (T.) antarctica*, USNM 97966); **d** *Thouarella (Euthorarella) hilgendorfi*, *Siboga* 251; **e** *Helicoprimnoa fasciola*, USNM 1180657 (holotype); **f** *Callozostron mirabile*, USNM 85294; **g** *Candidella imbricata*, USNM 57553; **h** *Plumarella penna*, USNM 1107503; **i** *Acanthoprimnoa goesi,* USNM 52968; **j** *Pterostenella plumatilis*, USNM 76964; **k** *Verticillata castellviae*, USNM 58167 (paratype); **l** *Tokoprymno maia*, USNM 81535 (holotype); **m** *Primnocapsa plumacea*, AM G12123 (holotype) from ZP & L-G, 2012); **n** *Heptaprimnoa patagonica*, USNM 1162059 (holotype); **o** *Parastenella spinosa*, USNM 98039; **p** *Faxiella abietina*, MCZ 4802 (holotype)

Clade 1B2 consists of two subclades. Both of the subclades of 1B2 were also found by Taylor & Rogers [3], but were based on fewer taxa. One subclade consists of two genera, *Candidella* and *Parastenella*, which are characterized by having polyps arranged perpendicular to the branch (Fig. 3g, o). The close relationship between these two taxa was also recovered by the morphology-based tree of Cairns & Bayer [2]. *Parastenella* has an autapomorphic character of having its marginals offset from its opercular scales (Fig. 3o). *Primnoa* and *Australogorgia*, members of the second sister subclade of 1B2, are united morphologically by having only six longitudinal rows of body wall scales, both of which were also recovered in clade D of the morphological tree of Cairns & Bayer [2]. *Loboprimnoa* Cairns, 2016 was described quite recently and thus not previously analyzed. This genus is very distinctive with several unique characters, including a lack of coenenchymal scales and a small sac-like body covered by transversely arranged scales (Fig. 2e). Its morphology is most similar to *Callozostron*, based on its extremely elongate marginal scales. However, this character may be convergent as *Callozostron* fell in the more distant clade 5B1 (relative to clade 1B2) of the molecular phylogeny (Fig. 1). The presence or absence of spurs on the basal scales of *Primnoa notialis* [2] does not seem to have taxonomic significance, and the inclusion of *Pyrogorgia* in this subclade is morphologically unexpected based on taxonomically relevant characters (see *Key to the genera and subgenera of the Primnoidae*). No molecular data was obtained for *Microprimnoa*. However, based on the provided key to the primnoid genera, this genus shares morphological similarities with *Loboprimnoa* and *Abyssoprimnoa*, and is, therefore, hypothesized to fall within clade 1B.

#### Clades 2, 3, and 4

**Fig. 4:**
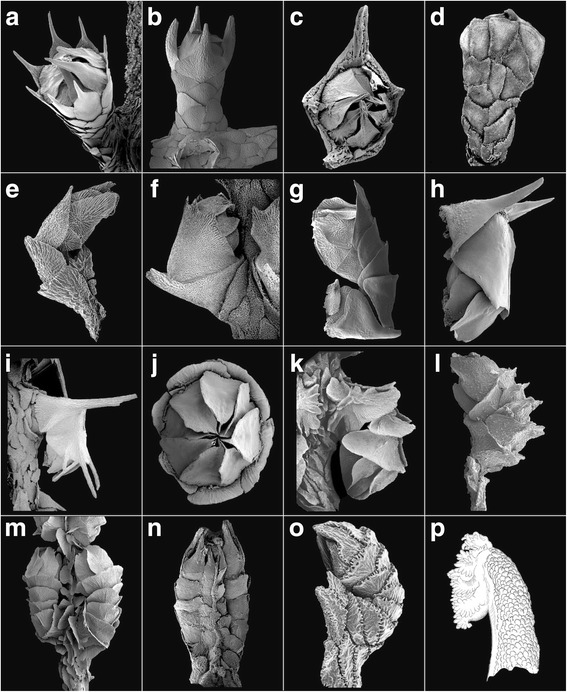
**a** *Mirostenella articulata*, USNM 84344; **b** *Pachyprimnoa asakoae*, USNM 1278861 (holotype); **c** *Dasystenella acanthina*, BM 1889.5.27.48; **d** *Primnoa resedaeformis*, USNM 16946; **e** *Perissogorgia viridis*, USNM 80043 (paratype); **f** *Calyptrophora japonica*, USNM 30027; **g** *Narelloides crinitus*, USNM 1180659 (holotype); **h** *Paracalyptrophora kerberti*, USNM 30145; **i** *Arthrogorgia ijimai*, USNM 30028; **j** *Australogorgia aldersladei*, NTM CO 13054 (holotype); **k** *Narella regularis*, USNM 49385 (neotype); **l** *Metanarella nannolepis*, USNM 1180661 (holotype); **m** *Paranarella watlingi*, USNM 1096721 (holotype); **n** *Artnzia gracilis*, USNM 78666; **o** *Callogorgia verticillata*, USNM 59107; **p** *Ophiodiogorgia paradoxa*, USNM 58165 (holotype)

Clade 2, consisting of only two genera, *Pachyprimnoa* (Fig. 4b) and one species of *Perissogorgia* (Fig. 4e), has no obvious morphological synapomorphies, and *Pachyprimnoa* was described recently [47] and has not been phylogenetically analyzed previously. Cairns [47] suggested that *Pachyprimnoa* was morphologically closest to *Candidella*. However, *Candidella* grouped with *Parastenella* spp. in a subclade of clade 1B2, a grouping that is consistent with Cairns & Bayer [2] who placed both of these genera in a subclade of their clade E.

Clade 3 consists of a single species, *Perissogorgia monile* Bayer & Stefani, 1989, and there appears to be no morphological reason to distinguish this clade (species) from adjacent clades or even from any of the other seven species of *Perissogorgia*. It should be noted that the type species of *Perissogorgia*, *P. viridis* Bayer & Stefani, 1989, was not included in this dataset, and it is suggested that analyses of the type and other species in this genus are needed to clarify the relationships among *Perissogorgia* species.

Clade 4 consists of two morphologically very similar genera, *Fanellia* and *Callogorgia*, which were also united in a morphoclade by Cairns & Bayer [2]. Once considered to be the same genus, Bayer [48] separated them based on differences in the texture of the outer surface of the body wall scales. However, the mixing of species from both purported genera in clade 4 suggests that they are more likely to be the same genus (Fig. 1, III). Therefore, as implied by [3, 26], the synonymy of *Fanellia* Gray, 1870 with its senior synonym *Callogorgia* Gray, 1858 (Fig. 4o) is proposed, resulting in a genus with approximately 34 species (see [2, 3]). Lastly, the inclusion of *Thouarella coronata* (Kinoshita, 1908) in this clade is morphologically inconsistent, and it is suggested that the taxonomic identification of this specimen (specimen code SW3809, from [3]) be reassessed, as this species is known from only two specimens (see Taxonomic revision in [13]). Consequently, more individuals of this species are needed to confirm its placement.

#### Clade 5

The remainder of the tree, clade 5 (Fig. 1), contains the majority of the primnoid genera. There appears to be no consistent morphological justification to distinguish clade 5A from 5B, except that all genera in clade 5A have polyps arranged “in whorls”, whereas those genera in clade 5B have a variety of arrangements, including as “in whorls”. However, the ancestral condition of the ancestor to clade 5 was most likely “in whorls” (Additional file 6: Figure S1b, see section *Evolution and Utility of Morphological Characters used for Taxonomy*), which suggests that the diversity of polyp arrangement observed in subclade 5B (“biserial” and “isolated, without order”) is secondarily derived.

Similarly, there appears to be no morphological distinction between clades 5A1 and 5A2. Clade 5A1 consists of two subclades, the first consisting of two genera: the monophyletic *Dasystenella* (Fig. 4c) and the monotypic *Heptaprimnoa* (Fig. 3n). The inclusion of one specimen of *Convexella magelhaenica* (Studer, 1879), which was not from this study (specimen FA78, [3]), in this subclade is unexpected, as other individuals of this species cluster more closely together in a subclade of 5A2. Moreover, in the original description of *Heptaprimnoa* [15], *Dasystenella* was one of four genera suggested as a potential sister genus based on morphology.

The other subclade in 5A1 consists of several species of *Metafannyella*, and also *Plumarella (Faxiella*) and *Aglaoprimnoa*, which invalidates the monophyly of the genus. *Aglaoprimnoa* is morphologically distinctive in having polyps with more than eight rows of poorly arranged body wall scales and in lacking an operculum (Fig. 2g); it is morphologically discrete and not particularly similar to *Metafannyella*. Similarly, *Plumarella (Faxiella*) is not morphologically similar to *Metafannyella* either. Although *Plumarella (Faxiella*) appears to be part of the genus *Metafannyella* in Fig. 1, morphologically it differs from that genus in lacking a circumoperculum and in lacking ascus body wall scales; *Metafannyella* contains both of these attributes. Moreover, the placement of *Plumarella (Faxiella*) is not close to the other three subgenera of this genus, and, consequently, argues for the elevation of this subgenus to genus level (Fig. 1, V). Although no molecular data are available for the genus *Scopaegorgia*, its morphology (see the *Diagnostic key to the genera and subgenera of the Primnoidae*) suggests that it is related to *Dasygorgia* and, therefore, is predicted to fall into clade 5A1.

Within the large and diverse clade 5A2 there are some problematic (para- and polypheletic) taxa but also some genera that were recovered as monophyletic, which validate their original morphological diagnoses, such as: *Arntzia, Ophidiogorgia, Primnoeides, Onogorgia, Digitogorgia* (only one species), and *Fannyella*, including all three of its subgenera (Fig. 2j, k, p), which were also monophyletic. Furthermore, the relatedness of *Fannyella* and *Onogorgia* was similarly revealed from morphology by Cairns & Bayer [2], based primarily on the synapomorphy of having body wall ascus scales (Fig. 1, IVb). One problematic polyphyletic genus is *Primnoella* (Fig. 2n). The species *P. insularis* Cairns, 2016, *P. divaricata* (Studer, 1879), and *P.* c.f. *delicatissima* Kükenthal, 1909, as well as *P. antarctica* Kükenthal, 1907 and *P. scotiae* Thomson & Richie, 1906 each grouped in disparate subclades, whereas *P. chiliensis* (Philippi, 1894) and a *Primnoella* sp. each paired with species of *Armadillogorgia* and *Ophidiogorgia*, respectively. Whereas the grouping of *P. antarctica* and *P. scotiae* as sister to a clade with species of *Convexella* can be explained morphologically [2], those species that group with *Armadillogorgia* and *Ophidiogorgia* do not exhibit any obvious morphological similarities. A more comprehensive sampling of *Primnoella* species and individuals is needed to more robustly assess the systematic status of this genus. *Plumarella (Verticillata*) (Zapata-Guardiola & López-González 2012) (Fig. 3k), in a circumstance similar to *Plumarella (Faxiella*) (clade 5A1), does not occur near the other subgenera of *Plumarella*, and thus should be elevated to the genus rank (Fig. 1, V). Furthermore, the placement of *Plumarella (Plumarella) pourtalesii* (Verrill, 1883) of this subclade was incongruent, as the other members of this subgenus occur more removed in clade 5B1. In addition, *Armadillogorgia* (Fig. 2b) was also recovered as polyphyletic based on three samples (one identified species, and two unidentified species).

There appears to be no morphological distinction between clades 5B1 and its sister clade that contains clades 5B2 and 5B3 (clades 5B2 and 5B3 were reconstructed as sister clades but this clade was poorly supported (BL = 0.81, ML = 39), and, therefore, was not given a clade label). Clade 5B1 consists of two smaller clades. The first subclade is composed of the monophyletic *Ainigmaptilon* and the polyphyletic *Callozostron*, which have little morphology in common. For example, *Ainigmaptilon* is pennatulacean-like and has its polyps arranged in polyp leaves (Fig. 2a). *Callozostron pinnatum*, the only species of this genus to have alternate pinnate branching [47], groups with members of *Plumarella* in the second subclade of 5B1. Nonetheless, the various species of *Plumarella* in subgenera *Plumarella* (*Plumarella*) and *Plumarell*a (*Dicholaphis)* form a monophyletic and paraphyletic clade, suggesting that *Dicholaphis* (differing only in having its polyps oriented randomly instead of biserially) should be synonymized with the nominate subgenus *Plumarella* (*Plumarella*) (Fig. 1, VI).

No molecular data were recovered for the genera *Helicoprimnoa*, *Acanthoprimnoa, Primnocapsa*, *Pseudoplumarella*, and *Pterostenella.* However, Cairns [47] suggested *Helicoprimnoa* to have a morphological affinity with *Plumarella*, and the included key to the Primnoidae places it close to *Callozostron*, both members of clade 5B1. Similarly, Cairns & Bayer [2] suggested a morphological resemblance of *Acanthoprimnoa* to *Plumarella* (clade 5B1), which is supported by the morphological cladogram of Cairns & Bayer [2] and the included key to the Primnoidae*.* The genus *Primnocapsa* shows a morphological resemblance to *Plumarella* and *Acanthoprimnoa* as suggested by the authors of the genus [49] and the included key to the Primnoidae. Therefore, it is hypothesized to fall in clade 5B1. Lastly, based on the provided key the primnoid genera *Pseudoplumarella* contains morphological similarities with *Armadillogorgia*, and *Pterostenella* with *Callozostron* and *Verticillata*. Therefore, *Pseudoplumarella* is hypothesized to fall in clade 5A2, and *Pterostenella* in either clade 5B1 or 5A2.

Clades 5B2 and 5B3 can be morphologically distinguished based on their number of rows of body wall scales. Clade 5B2 contains eight rows, and clade 5B3 contains six or seven rows. 5B2 consists of the paraphyletic *Tokoprymno* (Fig. 3l) and the monophyletic *Mirostenella* (Fig. 4a*)*, the latter differentiated by having a calcareous axis interrupted by organic nodes, and its polyps arranged in whorls (not biserially, see *Key to the genera and subgenera of the Primnoidae*).

Finally, clade 5B3 is reserved for the numerous and diverse species of the monophyletic genus *Thouarella*. Morphologically, this clade can be distinguished as having six rows of body wall scales. For the purpose of coding, the genus was divided into four groups to accommodate interpretation of variation in polyp placement and branching (Table 2). Our groups a and b equate to *Thouarella* group 1 (polyps isolated) sensu [3] and our groups c and d equate to their group 2 (polyps in whorls). As discussed previously, *T. coronata*, the only species analyzed in our group d, was placed in clade 4. The only taxon in our group c (group 2 of [3] is *T. laxa* Versluys, 1906, which branches sister to the rest of clade 5B3 and forms a monophyletic group characterized by having whorled polyps and pinnate branching. This group, as suggested by Taylor & Rogers [3], should be a separate subgenus, the earliest available name being *Thouarella (Euthouarella*) Kükenthal, 1915 (Fig. 1, VII; Fig. 4d). The remaining species, pertaining to our groups a and b (group 1 of [3]), grouped together and are characterized by having isolated polyps and bottlebrush branching. Containing the type species of the genus, *T. antarctica*, it should be the nominate subgenus (Fig. 3c). This clade includes two species identified as *Plumarella*: *P. diadema* (Cairns, 2006) and *P. undulata* (Zapata-Guardiola & López-González, 2010), both of which were originally placed in the genus *Thouarella*, but were subsequently transferred to *Plumarella* by Cairns [14] and Taylor & Rogers [3] because the two species did not have keeled marginal scales. But on re-analysis, both of these species were found to have multi-keeled marginal scales, unlike the traditional single keel of most other *Thouarella* species. Their placement firmly within the *Thouarella* clade, strongly suggests that a multi-keel marginal scale is homologous to the single keel, and thus these two species are reassigned to their original genus (Fig. 1, VIII).

### Evolution and utility of morphological characters used for taxonomy

Nine of the 23 morphological characters used by Cairns & Bayer [2] were mapped on the molecular tree in order to reconstruct ancestral states (Additional file 6: Figure S1a-i) and analyze the correspondence of morphological to molecular data. These characters were chosen for their value in discriminating genera in the morphology-based key (see *Key to the genera and subgenera of the Primnoidae*). At least one character state for all nine of the characters analyzed was reconstructed as convergent, and highlights a general pattern of independent evolution (i.e., homoplasy) among many primnoid characters (Additional file 6: Figure S1a-i). This pattern is consistent with other gorgonian octocorals that exhibit modular branching [23, 50], and it is hypothesized that this type of colony organization, and resulting lability, may increase the adaptability of the organism to its environment [50]. However, reversals were not as common as convergences. For example, four of the nine characters (colony shape, correspondence of opercular and marginal scales, ascus scales, and infrabasal scales) showed reversals to the root ancestral state (Additional file 6: Figure S1a, d, e, h), which suggests, at least, a relative amount of constraint on the evolution of some primnoid characters. Nonetheless, the frequency of independently-derived morphological similarities among distantly related lineages of primnoids implies that the developmental mechanism responsible for producing various morphologies, particularly colony shape, may not require extensive evolutionary improvisation. Instead, higher degrees of morphological change could be attributed to relatively smaller evolutionary modifications, such as changes in gene regulation [25].

Despite the high levels of homoplasy found among primnoids, some morphological characters were found to be useful for systematic evaluations. For example, coordination of polyps, presence of the ascus body wall scale, number of rows of body wall scales, and number of marginal scales were useful in distinguishing different clades, and helped define the taxa within them. The following discusses the results of each of the nine reconstructed characters.

#### Colony shape

The character states of colony shape (Table 2, character 1, and Additional file 6: Figure S1a) are routinely used in species and genus definitions, despite the fact that many genera (e.g., 16 of 44 as analyzed by [2]) have at least two character states, and, in the key to the genera included herein, four genera have been keyed twice or more times to account for multiple states of this character. Even if the four dichotomous states and two pinnate states are collapsed to only dichotomous and pinnate, there results a bewilderingly complex character state transformation series when analyzed on the molecular phylogeny (Additional file 6: Figure S1a). However, this is consistent with the results of the morphotree of Cairns & Bayer [2]: page 17), for which they concluded that this character “may not be of much use for determining phylogeny”. Nonetheless, colony branching is often consistent at the genus level, and can be useful in characterizing certain genera. In addition, colony form does help to characterize clades 1A and 1B (all having a form of dichotomous branching), and also helps to characterize the two subgenera of *Thouarella* [*Thouarella* (*Thouarella*) being bottlebrush, and *Thouarella* (*Euthouarella*) being primarily pinnate and dichotomous].

The ancestral state of colony shape of the common ancestor of the Primnoidae was found to be the “dichotomous planar” mode by both maximum likelihood (ML = 0.98) and parsimony reconstructions (Additional file 6: Figure S1a; Fig. 1, I). Similar to other octocorallian groups such as shallow-water plexaurids and gorgoniids [22] and deep-sea isidids [23], branching morphology in primnoids appears to be highly labile. In this analysis, individual states were gained independently multiple times, and, therefore, convergence in colony shape appears to be common. Reversals to ancestral states were also found, but occurred less frequently. Examples of a reversal to the root ancestral state of “dichotomous planar” were found in *Mirostenella* (clade 5B2) from the derived states of “unbranched” (ML = 0.79, ancestor to clade 5B) and “dichotomous bushy” (ML = 0.96, ancestor to clade 5B2), *Metafannyella* (clade 5A1) from the derived state of “unbranched” (ML = 0.76, ancestor to clade 5A), and *Fannyella* (subclade in clade 5A2) from the derived states of “unbranched” (ML = 0.98, ancestor to clade 5A2) and “dichotomous lyriform” (ML = 0.79, ancestor to subclade within 5A2) (Additional file 6: Figure S1a).

#### Coordination of polyps

The root ancestral character state of “coordination of polyps” (Table 2, character 2; Additional file 6: Figure S1b) was reconstructed as “in whorls” (Figs. 2i, k, p) with parsimony and maximum likelihood (ML = 0.99), from which all other states evolved, some of them multiple times. This is consistent with the interpretation of the morphology-based phylogeny of Cairns & Bayer [2]. Nonetheless, this character gives little cladistic support to the tree, only helping to distinguish between the two subgenera of *Thouarella*, each with either polyps “in whorls” or “isolated, without order”. However, the subgenus *Plumarella (Dicholaphis)*, which has polyps “isolated, without order” did not group with members of *Thouarella* that contain polyps in this state (clade 5B3) [or *Primnoa* species that are also in this state (clade 1B2)], but, instead, in a clade with members of *Plumarella* (*Plumarella*) that contain “biserial” polyps, and whose common ancestor was reconstructed as “biserial” with both methods (ML = 0.99) (Additional file 6: Figure S1b, subclade of 5B1). Consequently, polyp arrangement in *Plumarella* (*Dicholaphis*) is mostly likely convergent, and is herein synonymized with the nominate subgenus *Plumarella* (*Plumarella*) (Fig. 1, VI).

#### Operculum

The root ancestral state of the character “operculum” (Table 2, character 3; Additional file 6: Figure S1c) (Figs. 3m, 4j) is the presence of an operculum (ML = 0.99), from which it is lost independently (Fig. 2b) in four linages, all within clade 5A (Additional file 6: Figure S1c). This is quite different from the morphology-based phylogeny of Cairns & Bayer [2] in which the ancestral state was assumed to be the absence of an operculum, and the acquisition of an operculum occurs but once. Nevertheless, this character appears to provide no utility as a synapomorphy that unites the species of more than one genus.

#### Correspondence of opercular and marginal scales

The root ancestral state of the character “correspondence of opercular and marginal scales” (Table 2, character 4; Additional file 6: Figure S1d) was the absence of such a correspondence (ML = 0.96), characterized by those genera having two, four, five, or six marginal scales, or eight scales that are not aligned with the operculars. Thus, this character is somewhat linked to the character “number of marginal scales” (character 9). The tree suggests a transformation from the root ancestral state of “no correspondence” to “correspondence” four times (clades 1A, 1B2, 2, and 3A; Additional file 6: Figure S1d), and a reversal to the ancestral state twice (clades 1B2 and 5A1), as well as a transformation to the offset condition (clade 1B2) (Fig. 3o). The various number of opercular scales other than eight help to characterize clades 1A and 1B1 (Fig. 1, II). Although the ancestral state of this character was found to be the correspondence state by Cairns & Bayer [2], the character states implied by their morphology-based tree show the same relative relationship of states.

#### Ascus scales

The root ancestral state of the character “ascus scales” (presence or absence of ascus body wall scales: Table 2, character 5; Additional file 6: Figure S1e) was the absence of such scales (ML = 0.99), with a transformation to the presence of ascus body wall scales twice (clades 5A1 and 5A2; Additional file 6: Figure S1e). In clade 5A2, the presence of ascus body wall scales was reconstructed as the ancestral state of the ancestor of a subclade that unites members of *Onogorgia* and *Fannyella* as sister taxa (ML = 0.91), both of which maintain this state in extant members. However, in clade 5A1, transformation to the presence of ascus body wall scales was found among species of *Metafannyella* that grouped paraphyletically in a subclade with *Aglaoprimnoa stefanii* Bayer, 1996 and *Plumarella* (*Faxiella*) *delicatula* (Thomson & Rennet, 1931), taxa that do not share this character state. The “true” or well-developed typical ascus scales are those found in *Onogorgia* and *Fannyella* (clade 5A2), with those of *Metafannyella*, as described by Cairns & Bayer [2], being much reduced in structure. The morphology-based tree of Cairns & Bayer [2] indicates a relatively close phylogenetic relationship among *Onogorgia*, *Fannyella*, and *Metafannyella*. However, in light of the molecular phylogeny presented here (Fig. 1, IV), and the reconstruction of the ancestral states of “ascus body wall scales” among these taxa, it is suggested that the “weak” ascus scales of *Metafannyella* are not homologous to the “true” ascus scales in the sense of those of *Onogorgia* and *Fannyella*. Instead, they more likely represent the independent (i.e., convergent) evolution of a morphologically similar character.

#### Number of longitudinal rows of body wall scales

The root ancestral state of the character “number of longitudinal rows of body wall scales” (Table 2, character 6; Additional file 6: Figure S1f) was recovered as “two” (ML = 0.90). This state is not in accordance with the interpretation of Cairns & Bayer [2] based on morphology, which suggested the “lack of arrangement” as the ancestral state. This ambiguity is understandable given the intricacy of this character. For example, various independent appearances of several of the character states are found throughout the tree. Most notably, the number of longitudinal rows of body wall scales of “six”, “five”, and “not arranged in rows” appear to have evolved multiple times in primnoids, which, consequently, reduces the usefulness of this character for systematic purposes. Nevertheless, as with characters 4 (correspondence of opercular and marginal scales), 7 (number of scales in each abaxial body wall row or abaxial face) and 9 (number of marginal scales) [see below for 7 and 9], this character helps to define members of clades 1A and 1B1 (Fig. 1, II).

#### Number of scales in each abaxial body wall row or abaxial face

The root ancestral state of the character “number of scales in each abaxial body wall row or abaxial face” (Table 2, character 7; Additional file 6: Figure S1 g) was recovered as “variable, but usually over 5” scales (ML = 0.96). This ancestral character state was the same as that suggested by Cairns & Bayer [2], but at that time no genera were known that had the state of “no abaxial body wall scales”. The recovered ancestral state of “variable, but usually over 5” (Fig. 2n) suggests, somewhat counterintuitively, that the three other character states pertaining to the number of “fixed” scales (Fig. 4e-m) in the abaxial rows (i.e., character states of 2, 3/4, and 5 scales), are derived from an ancestral condition of a variable number of over five scales, which in at least two species (*Abyssoprimnoa gemina* Cairns, 2015 and *Loboprimnoa exotica* Cairns, 2015) further transform to a state that lacks body wall scales altogether. As with characters 4 (correspondence of opercular and marginal scales), 6 (number of longitudinal rows of body wall scales), and 9 (number of marginal scales) [see below for 9], this character helps to define members of clades 1A and 1B1 (Fig. 1, II).

#### Infrabasal scales

The root ancestral state of the character “infrabasal scales” (presence or absence of infrabasal scales: Table 2, character 8; Additional file 6: Figure S1 h) is the absence of such scales (ML = 0.97). The presence of infrabasal scales is rare among the primnoid genera; however, it was not recovered as a synapomorphy unique to any clade. It appears to have evolved twice (clades 1A and 1B1; Additional file 6: Figure S1 h), yet helps to define most of the genera in clade 1A, and is present in one genus (*Abyssoprimnoa gemina*) in clade 1B1. The morphology-based tree of Cairns & Bayer [2] also assumes the same ancestral state, and groups *Calyptrophora* and *Paracalyptrophora* in a monophyletic clade, similar to that of clade 1A.

#### Number of marginal scales

The character “number of marginal scales” (Table 2, character 9; Additional file 6: Figure S1i), which is most commonly eight (Fig. 4a), can also occur in states of two (Fig. 4f), four (Fig. 4b), five (Fig. 4c), six (Fig. 4j) and more than eight (Fig. 2g). The recovered ancestral state for this character at the root node was equivocal, being a mixture of two, four, and eight scales with parsimony, and four (ML = 0.33) and eight (ML = 0.56) with maximum likelihood. The morphological tree of Cairns & Bayer [2] recovered eight as the ancestral number of marginal scales [the state that received the highest ML probability (ML = 0.56) in this study]. The relative uncertainty of the root common ancestor with this character makes it difficult to trace its evolution with confidence. However, several of the other states do appear to have clearly evolved more than once (e.g., 5 and more than 8 marginal scales). In addition, and similar to characters 4 (correspondence of opercular and marginal scales), 6 (number of longitudinal rows of body wall scales), and 7 (number of scales in each abaxial body wall row or abaxial face), this character helps to define members of clades 1A (those taxa with 2 marginal scales, and 1B1 (those taxa with 4 marginal scales) (Fig. 1, II).

### Key to the genera and subgenera of the Primnoidae

The first key to the primnoid genera was generated by Kükenthal [11], with later modifications in 1919 [51] and 1924 [52]. Bayer [53] published the first illustrated key to the genera, which was elaborated in Bayer [54] in the context of a key to all octocorallian genera. Bayer & Stefani [55] produced another key in French, and, ultimately, a key to the 36 known primnoid genera was published by Cairns & Bayer [2]. The key presented herein, including 43 genera and five non-nominate subgenera, is a modified version of the Cairns & Bayer [2] key. It includes 10 newly described genera, two newly described subgenera, the synonymy of three genera (*Tauroprimnoa, Fanellia* and *Amphilaphis*), the synonymy of the subgenus *Dicholaphis*, and a re-evaluation of the subgenera of the genus *Thouarella*. Although it represents an improvement over the 2009 key, based on the rapid rate of species and genus discoveries, it is anticipated that it may soon become outdated. It is now estimated that there are 279 valid primnoid species as of January 1, 2017 [4]. The definitions and illustrations of the morphological terms used in this key can be found in [56].

## Diagnostic key to the genera and subgenera of the Primnoidae

I. Polyps united in groups forming polyp-leaves placed along axis as in some pennatulaceans: ***Ainigmaptilon*** (Fig. 2a)
II. Polyps individually distinct, or united basally, but not united in groups forming polyp-leaves.
  A. Polyps adnate to coenenchyme except for oral region.
    1. Colonies dichotomous, large and robust, terminal branches long and flexible; polyps large, arranged in close-set whorls; abaxial side covered by two rows of narrow, sickle-blade-shaped sclerites; distalmost polyp scales not differentiated as operculum: ***Armadillogorgia*** (Fig. 2b)
    2. Colonies closely pinnate, slender and plumose, side-branches short and stiff; polyps small, not in whorls, biserial or in close spirals, directed obliquely upward; abaxial side with only one longitudinal row of scales, adaxial side extremely short and adnate to coenenchyme, lacking scales below marginals; operculum well developed, tall, conical, the triangular opercular scales fitting closely together: ***Pseudoplumarella*** (Fig. 2c)
  B. Polyps not adnate to coenenchyme (e.g., appressed, inclined, or perpendicular)
    1. Polyps having body wall sclerites in the form of thick plates, not imbricating but closely fitted as in mosaic, not aligned in regular rows; coenenchymal scales present: ***Microprimnoa*** (Fig. 2d)
    2. Polyps having body wall sclerites in the form of flattened non-imbricate transversely arranged rods covering a sac-like body; coenenchymal scales absent: ***Loboprimnoa*** (Fig. 2e)
    3. Polyps lacking body wall scales, having only four marginal scales; coenenchymal scales present: ***Abyssoprimnoa*** (Fig. 2f)
    4. Polyps having body wall sclerites in the form of scales, thin or thick, clearly imbricating and aligned in regular rows at least on immature polyps; coenenchymal scales present
      - Polyps having sclerites aligned in five to eight complete, well-developed rows on all sides of polyp, at least on immature polyps, resulting in adaxial side of polyp being completely covered with scales.
        1^1^ Sclerites of mature polyps in multiple (more than eight) rows, the longitudinal alignment of eight regular rows present only in immature polyps; distalmost scales not differentiated as a well-organized operculum.
          a^1^ Colonies robust, dichotomous; polyps large, curved inward toward axis; polyps arranged in whorls; numerous distal body scales with strong apical keel: ***Aglaoprimnoa*** (Fig. 2g)
          b^1^ Colonies small, delicate, pinnate; polyps small, not curved inward; polyps arranged biserially (not in whorls); distal body scales lack keel but close over retracted tentacles and mouth: ***Primnoeides*** (Fig. 2h)
        2^1^ Sclerites of polyps in five to eight longitudinal rows; distalmost scales differentiated as an operculum.
          a^1^ Marginal scales of polyps form a circumoperculum that folds over bases of opercular scales.
            1^2^ Outer surface of abaxial and lateral body scales, including marginals and submarginals, with a well-defined, transverse, serrate, spinose, or granular ridge extending across the greatest width of the sclerite, separating the exposed distal part from the proximal part covered by the distal margin of the next lower scale. The transverse ridge is continuous with lateral and distal margins and forms a shallow concavity (the ascus scale) on upper surface of sclerite.
              a^2^ Exposed outer surface of body wall scales sculptured with a serrate or spinose transverse ridge; inner surface of opercular scales ridged.
                1^3^ Colonies flagelliform; marginal scales without apical spine: ***Onogorgia*** (Fig. 2i)
                2^3^ Colonies branched; marginal and sometimes submarginal scales with a strong, smooth apical spine.
                 a^3^ Colonies bottle-brush shaped, with numerous simple twigs arising from all sides of main stems: ***Fannyella*** **(*Scyphogorgia*)** (Fig. 2j)
                 b^3^ Colonies dichotomously to quasi-pinnately branched: ***Fannyella*** **(*Cyathogorgia*)** (Fig. 2k)
              b^2^ Exposed outer surface of body scales sculptured with low, smooth projections and distinguished from the covered portion by a transverse row of granules or tubercles along a more or less thickened boundary between exposed and concealed part of scale; inner surface of opercular scales with a strong apical keel most prominent on abaxial and outer-laterals: ***Metafannyella*** (Fig. 2l)
            2^2^ No distinct boundary separating exposed distal part of body scales from proximal part covered by scale below.
              a^2^ Colonies flagelliform (unbranched), sometimes unattached, branch cross section round: ***Convexella*** (Fig. 2m)
              b^2^ Colonies flagelliform; branch cross section compressed: ***Primnoella*** (Fig. 2n)
              c^2^ Colonies abundantly branched; branch cross section round.
                1^3^. Polyps with eight rows of body wall scales.
                 a^3^. Branching dichotomous.
                  1^4^. Body wall scales radially ridged (not ascus-shaped): ***Pyrogorgia*** (Fig. 2o)
                  2^4^. Body wall scales as ascus in shape: ***Fannyella*** **(*Fannyella*)** (Fig. 2p)
                 b^3^. Branching as a bottlebrush
                  1^4^. Body wall scales smooth; operculars coarsely serrate distally; accessory opercular scales: ***Digitogorgia*** (Fig. 3a)
                  2^4^. Polyps in whorls; body wall scales smooth; marginals lacking keels, seven in number: ***Scopaegorgia*** (in part) (Fig. 3b)
                2^3^. Polyps with six to eight rows of body wall scales, the two adaxial rows having smaller and fewer scales especially near polyp base, these rows being covered by broadened adjacent inner laterals or naked: ***Thouarella*** sensu *lato*
                 a^3^. Polyps isolated on branches: ***Thouarella*** (***Thouarella***) (Fig. 3c)
                 b^3^. Polyps arranged in whorls or pairs: ***Thourella*** (***Euthouarella***) (Fig. 3d)
          b^1^. Marginal scales of polyps do not fold over bases of opercular scales.
            1^2^. Colonies unbranched or very sparsely branched.
              a^2^. Polyps with eight marginal scales; polyps fused basally.
                1^3^. Polyps linearly arranged in clusters on one side of branch; colony spirals (coiled); marginal spines not spinose: ***Helicoprimnoa*** (Fig. 3e)
                2^3^. Polyps arranged in whorls; colony not coiled; marginal scales highly spinose: ***Callozostron*** (in part: unbranched species) (Fig. 3f)
              b^2^. Polyps with four marginal scales; polyp bases not fused: ***Candidella*** (in part: unbranched species) (Fig. 3g)
            2^2^. Colonies abundantly branched.
              a^2^. Branching pinnate.
                1^3^. Polyps biserial or isolated, directed strongly upward.
                 a^3^. Distal edges of body wall scales spinose or serrate; inner face of sclerites tuberculate: ***Plumarella*** (in part: pinnately branched species) (Fig. 3h)
                 b^3^. Distal edges of body wall scales pectinate; inner faces of sclerites smooth (not tuberculate): ***Acanthoprimnoa*** (in part: pinnately branched species) (Fig. 3i)
                2^3^. Polyps in whorls, directed weakly upward.
                 a^3^. Body scales in five longitudinal rows; five marginal scales: ***Pterostenella*** (Fig. 3j)
                 b^3^. Body wall scales in eight longitudinal rows; eight marginal scales.
                  1^4^. Submarginal body wall scales not spinose: ***Verticillata*** (Fig. 3k)
                  2^4^. Submarginal body wall scales highly spinose: ***Callozostron*** (in part: pinnately branched species) (Fig. 3f)
              b^2^. Branching dichotomous or bottlebrush.
                1^3^. Eight marginal scales.
                 a^3^. Polyps arranged biserially.
                  1^4^. Inner surface of operculars keeled; brood polyps common: ***Tokoprymno*** (Fig. 3l)
                  2^4^. Inner surface of operculars not keeled; brood polyps rare.
                   a^4^. Marginals and operculars offset: ***Primnocapsa*** (Fig. 3m)
                   b^4^. Marginals and operculars aligned.
                    1^5^. Distal edges of body wall scales spinose or serrate; inner face of sclerites tuberculate: ***Plumarella*** (in part: dichotomously branched species) (Fig. 3h)
                    2^5^. Distal edges of body wall scales pectinate; inner faces of sclerites smooth (not tuberculate): ***Acanthoprimnoa*** (in part: dichotomously branched species) (Fig. 3i)
                 b^3^. Polyps arranged in whorls, pairs, or isolated
                  1^4^. Polyps isolated: ***Plumarella*** (in part: species with isolated
                  2^4^. Polyps arranged in pairs or whorls.
                   a^4^. Only seven rows of body wall scales: ***Heptaprimnoa*** (Fig. 3n)
                   b^4^. Eight rows of body wall scales.
                    1^5^. Marginal scales offset from opercular scales: ***Parastenella*** (Fig. 3o)
                    2^5^. Marginals aligned with opercular scales.
                     a^5^. Polyps paired; operculars keeled: ***Faxiella*** (Fig. 3p)
                     b^5^. Polyps whorled; operculars smooth.
                      1^6^. Organic nodes present at branch bifurcations; opercular scales not spinose: ***Mirostenella*** (Fig. 4a)
                      2^6^. Organic nodes lacking; marginal and opercular scales highly spinose: ***Callozostron*** (in part: dichotomously branched species) (Fig. 3f)
                2^3^. Four, five, or seven marginal scales.
                 a^3^. Four marginal scales; colonies uniplanar dichotomous.
                  1^4^. Marginal scales thin, with a straight distal margin; opercular scales uniform in size: ***Candidella*** (in part: branched species) (Fig. 3g)
                  2^4^. Marginal scales massive (thick) and pointed; opercular scales dimorphic in size: ***Pachyprimnoa*** (Fig. 4b)
                 b^3^. Five marginal scales; colonies bottlebrush in shape: ***Dasystenella*** (Fig. 4c)
                 c^3^. Seven marginals; colonies bottlebrush in shape: ***Scopaegorgia*** (in part) (Fig. 3b)
      - Polyps having sclerites aligned in complete, well-developed rows only on abaxial and sometimes outer-lateral sides, the adaxial side having a few, small (vestigial) or no (naked) sclerites below the adaxial marginal scales.
        1^1^. Abaxial side of polyps protected by large scales; distalmost eight scales form a well-differentiated operculum.
          a^1^. Polyps crowded irregularly around stems, not in regular whorls: ***Primnoa*** (Fig. 4d)
          b^1^. Polyps arranged in pairs or whorls around stems.
            1^2^. Abaxial body wall scales arranged in a single longitudinal row of large scales: ***Perissogorgia*** (Fig. 4e)
            2^2^. Abaxial body wall scales arranged in two longitudinal rows.
              a^2^. Body wall scales in two rows (no adaxial, or outer- and inner-lateral rows of scales, although one pair of outer- and inner-lateral marginal scales may be present).
                1^3^. Two pairs of large abaxial body wall scales.
                 a^3^. Both pairs of abaxial plates extend around body as a solid ring, the members inseparably fused along abaxial and adaxial symphysis: ***Calyptrophora*** (Fig. 4f)
                 b^3^. Abaxial plates extend around body but are not solidly fused along abaxial and adaxial symphysis.
                  1^4^. Colonies unbranched; a single medial infrabasal scale at base of polyp, six buccal scales: ***Narelloides*** (Fig. 4g)
                  2^4^. Colonies branched; one or more transverse rows of infrabasal scales; two buccal scales.
                   a^4^. One pair of infrabasal scales: ***Paracalyptrophora*** (Fig. 4h)
                   b^4^. Two or more transverse rows of infrabasal scales: ***Arthrogorgia*** (Fig. 4i)
                2^3^. Three to five pairs of abaxial body wall scales.
                 a^3^. Three or four pairs of abaxial scales enclose body; no inner- or outer-lateral scales.
                  1^4^. Six marginal scales; polyps arranged unilinearly and perpendicular to branch ***Australogorgia*** (Fig. 4j)
                  2^4^. Four marginal (buccal) scales (including two small adaxial scales); polyps arranged in downward pointing whorls: ***Narella*** (Fig. 4k)
                  3^4^. Three unpaired buccal scales; polyps arranged in downward pointing whorls: ***Metanarella*** (Fig. 4l)
                 b^3^. Five pairs of abaxial body wall scales; one pair of both inner- and outer- laterals present: ***Paranarella*** (Fig. 4m)
              b^2^. Body scales in four, six or eight longitudinal rows, the adaxial scales much smaller or even absent, and fewer in number, resulting in a naked adaxial region basally.
                1^3^. Colonies unbranched; polyps stand perpendicular to branch: ***Arntzia*** (Fig. 4n)
                2^3^. Colonies branched (pinnate, dichotomous); polyps appressed to branch, inclined upward: ***Callogorgia*** (Fig. 4o)
        2^1^. Abaxial side of polyps covered by numerous small warty plates not aligned in regular longitudinal rows except in small, immature individuals; distalmost sclerites not differentiated as opercular scales: ***Ophidiogorgia*** (Fig. 4p)

## Conclusions

The topology of the primnoid phylogeny recovered in this study (Fig. 1) was largely similar to the phylogeny of Taylor & Rogers [3] with the exception of the branching of the three principal subclades within clade 5 (i.e., clades 5A1 and 5A2 were sister), and the lack of statistical support to unite the subclades of Taylor & Rogers’ [3] clade 1. Twelve additional genus-level taxa, and, in some cases, additional specimens of congenerics were added in this dataset. Although genetic data for 10 of the 43 currently described genera were not able to be generated, hypotheses were made regarding the most likely phylogenetic placement of these genera based on morphology. Therefore, the relationships among some of the primnoid lineages recovered in this phylogeny may change with the addition of these genera.

The primary aims of this study were to reconcile the recovered molecular phylogeny to the morphology-based phylogeny of Cairns & Bayer [2], and to evaluate the evolution and utility of morphological characters commonly used in primnoid taxonomy. In regard to the former, there are many differences between the two phylogenies. However, there are also many morphological characters that support the various larger and smaller clades of the recovered molecular phylogeny. For example, morphological characters 4 (correspondence of opercular and marginal scales), 6 (number of longitudinal rows of body wall scales), 7 (number of scales in each abaxial body wall row or abaxial face) and 9 (number of marginal scales) all help to define clades 1A and 1B (Fig. 1, II); character 2 (coordination of polyps) helps to distinguish the two subgenera of *Thouarella* (Fig. 1, VII); the morphology-based subgenera of *Fannyella* are united in cohesive subclades; and, Cairns [47] accurately predicted the relatedness of *Heptaprimnoa* and *Dasystenella* based on morphology.

Although the results of this study largely focus on generic-level associations, the recovered phylogeny also raises systematic questions at the species level. For example, several individuals of the same species (e.g., *Calytrophora wyvillei* (clade 1A1), *Parastenella spinosa* (clade 1B2), and *Dasystenella acanthina* (clade 5A1) grouped paraphyletically or contained intraspecific genetic distances that could be consistent with different species or genera [34]. Datasets that are properly sampled for phylogeographic and/or population genetic questions will be needed to properly quantify the variation within these and other primnoid species.

### Taxonomic revision summary

The placement of various genera in the molecular phylogeny, and analyses of ancestral character states forced a re-evaluation of various taxa and the characters that define them. This, in turn, resulted in taxonomic rearrangements, and/or a reassessment of the taxonomic significance of a character. For example, the weak ascus scale of *Metafannyella* is re-interpreted as not being homologous to the ascus scale of *Onogorgia* and *Fannyella*, which results in this character being a synapomorphy for a clade that unites these two genera as sister taxa (Fig. 1, IV).

Taxonomic rearrangements include (1) the resurrection of the subgenus *Thouarella* (*Euthouarella*) (sensu [13]) and the nominate subgenus *Thouarella* (*Thouarella*) within the genus *Thouarella*, confirmed by the consistent placement of polyps in either whorls or an isolated arrangement (Fig. 1, VII); (2) the synonymy of the morphologically similar genera *Fanellia* and *Callogorgia*, implying that the tuberculate body wall scale ornamentation of *Fanellia* is not of generic importance (Fig. 1, III); (3) *Thouarella diadema* and *T. undulata*, previously transferred to *Plumarella*, are now replaced in *Thouarella* (as originally described) based on the re-evaluation that a multi-keeled inner marginal is homologous to the single-keeled inner marginal (Fig. 1, VIII); and the polyphyly of the subgenera of *Plumarella* resulted in (4) the elevation of *Plumarella* (*Faxiella)* and *Plumarella* (*Verticillata*) to generic status (e.g., now *Faxiella delicatula* and *Verticillata castellviae*) (Fig. 1, V) and (5) the synonymy of one of them *Plumarella* (*Dicholaphis*) with the nominate subgenus *Plumarella* (*Plumarella*) (Fig. 1, VI).

Our understanding of the phylogeny of the Primnoidae is increasing as more specimens are collected and more molecular analyses are performed using an increasing number of genes. The older phylogenies and evolutionary scenarios based on morphology (e.g.*,* [2, 51]) form a basis for comparison, and often complement the molecular phylogenies, but just as often do not. As the size of the primnoid phylogeny increases, the use of museum specimens (i.e., specimens that may have been preserved in alcohol for long periods of time) will likely be essential, as many of the primnoid genera are extremely rarely collected. Therefore, future comprehensive primnoid phylogenies, coupled with resulting taxonomic rearrangements, may depend on the use of types and non-types from museum collections.

## Additional files

Additional file 1: **Table S1A** and **S1B.** Specimens sequenced with taxon name, sample ID, collection location, collection station, year collected, preservation type, type specimen information, and Genbank Accession numbers. (XLSX 27 kb)

Additional file 2: Nexus alignments using partitions and models chosen by either BIC or AIC by the program Partition Finder. (XLSX 106 kb)

Additional file 3: **Table S2.** Data block assignments and Partition Finder results. Partition schemes and molecular models of evolution chosen by either AIC (Akaike Information Criterion) or BIC (Bayesian Information Criterion). (XLSX 36 kb)

Additional file 4: **Table S3.** Character state assignments for each genus or subgenus species-group from characters listed in Table 2. Numbers in parentheses correspond character designations from Cairns & Bayer [2]. (XLSX 11 kb)

Additional file 5: **Table S4.** Outputs of ancestral state reconstructions using maximum likelihood for each character and for all nodes. (XLSX 126 kb)

Additional file 6: **Figure S1a-i.** Phylogenies with mapped ancestral state reconstructions using maximum parsimony and select maximum likelihoods (see text) for each morphological character. (PDF 1095 kb)
