# Supplementary material for: A phylogenetic analysis of the Primnoidae (Anthozoa: Octocorallia: Calcaxonia) with analyses of character evolution and a key to the genera and subgenera
Source: BMC Evol Biol. 2018 May 2;18:66. doi: 10.1186/s12862-018-1182-5 (PMC5930830; doi:10.1186/s12862-018-1182-5)
Supplement: Supplementary file 6 — Figure S1a-i. Phylogenies with mapped ancestral state reconstructions using maximum parsimony and select maximum likelihoods (see text) for each morphological character. (PDF 1095 kb) [file 12862_2018_1182_MOESM6_ESM.pdf]

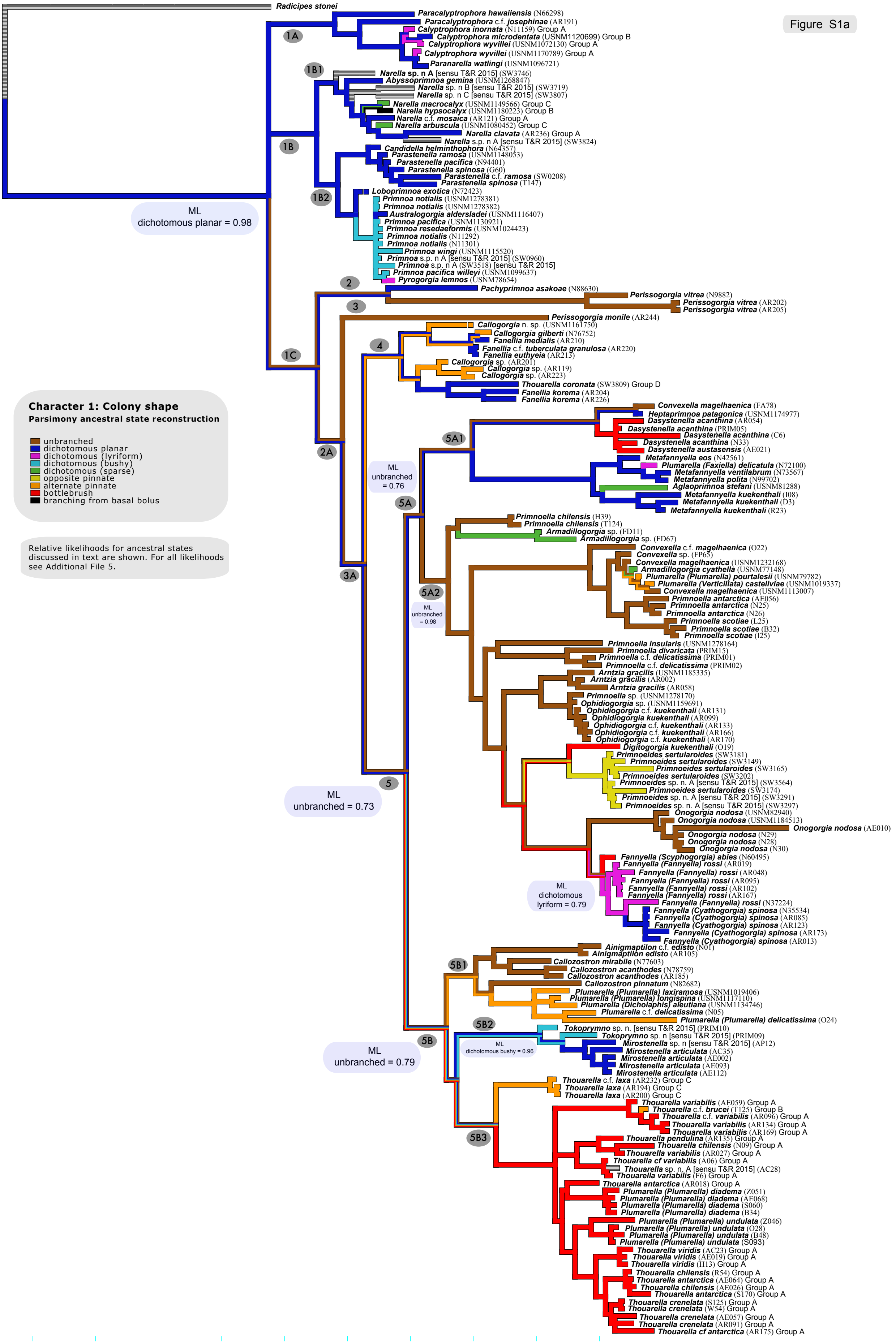
Radicipes stonei
Paracalyptrophora hawaiiensis (N66298)
Paracalyptrophora c.f. josephinae (AR191)
Calyptrophora inornata (N11159) Group A
Calyptrophora microdentata (USNM1120699) Group B
Calyptrophora wyvillei (USNM1072130) Group A
Calyptrophora wyvillei (USNM1170789) Group A
Paranarella watlingi (USNM1096721)
Narella sp. n A [sensu T&R 2015] (SW3746)
Abyssoprimnoa gemina (USNM1268847)
Narella sp. n B [sensu T&R 2015] (SW3719)
Narella sp. n C [sensu T&R 2015] (SW3807)
Narella macrocalyx (USNM1149566) Group C
Narella hypsocalyx (USNM1180223) Group B
Narella c.f. mosaica (AR121) Group A
Narella arbuscula (USNM1080452) Group C
Narella clavata (AR236) Group A
Narella s.p. n A [sensu T&R 2015] (SW3824)
Candidella helminthophora (N64357)
Parastenella ramosa (USNM1148053)
Parastenella pacifica (N94401)
Parastenella spinosa (G60)
Parastenella c.f. ramosa (SW0208)
Parastenella spinosa (T147)
Loboprimnoa exotica (N72423)
Primnoa notialis (USNM1278381)
Primnoa notialis (USNM1278382)
Australogorgia aldersladei (USNM1116407)
Primnoa pacifica (USNM1130921)
Primnoa resedaeformis (USNM1024423)
Primnoa notialis (N11292)
Primnoa notialis (N11301)
Primnoa wingi (USNM1115520)
Primnoa s.p. n A [sensu T&R 2015] (SW0960)
Primnoa s.p. n A (SW3518) [sensu T&R 2015]
Primnoa pacifica willeyi (USNM1099637)
Pyrogorgia lemnos (USNM78654)
Pachyprimnoa asakoae (N88630)
Perissogorgia vitrea (N9882)
Perissogorgia vitrea (AR202)
Perissogorgia vitrea (AR205)
Perissogorgia monile (AR244)
Callogorgia n. sp. (USNM1161750)
Callogorgia gilberti (N76752)
Fanellia medialis (AR210)
Fanellia c.f. tuberculata granulosa (AR220)
Fanellia euthyeia (AR213)
Callogorgia sp. (AR201)
Callogorgia sp. (AR119)
Callogorgia sp. (AR223)
Thouarella coronata (SW3809) Group D
Fanellia korema (AR204)
Fanellia korema (AR226)
Convexella magelhaenica (FA78)
Heptaprimnoa patagonica (USNM1174977)
Dasystenella acanthina (AR054)
Dasystenella acanthina (PRIM05)
Dasystenella acanthina (C6)
Dasystenella acanthina (N33)
Dasystenella austasensis (AE021)
Metafannyella eos (N42561)
Plumarella (Faxiella) delicatula (N72100)
Metafannyella ventilabrum (N73567)
Metafannyella polita (N99702)
Aglaoprimnoa stefani (USNM81288)
Metafannyella kuekenthali (I08)
Metafannyella kuekenthali (D3)
Metafannyella kuekenthali (R23)
Primnoella chilensis (H39)
Primnoella chilensis (T124)
Armadillogorgia sp. (FD11)
Armadillogorgia sp. (FD67)
Convexella c.f. magelhaenica (O22)
Convexella sp. (FP65)
Convexella magelhaenica (USNM1232168)
Armadillogorgia cyathella (USNM77148)
Plumarella (Plumarella) pourtalesii (USNM79782)
Plumarella (Verticillata) castellviae (USNM1019337)
Convexella magelhaenica (USNM1113007)
Primnoella antarctica (AE056)
Primnoella antarctica (N25)
Primnoella antarctica (N26)
Primnoella scotiae (L25)
Primnoella scotiae (B32)
Primnoella scotiae (I25)
Primnoella insularis (USNM1278164)
Primnoella divaricata (PRIM15)
Primnoella c.f. delicatissima (PRIM01)
Primnoella c.f. delicatissima (PRIM02)
Arntzia gracilis (USNM1185335)
Arntzia gracilis (AR002)
Arntzia gracilis (AR058)
Primnoella sp. (USNM1278170)
Ophidiogorgia sp. (USNM1159691)
Ophidiogorgia c.f. kuekenthali (AR131)
Ophidiogorgia kuekenthali (AR099)
Ophidiogorgia c.f. kuekenthali (AR133)
Ophidiogorgia c.f. kuekenthali (AR166)
Ophidiogorgia c.f. kuekenthali (AR170)
Digitogorgia kuekenthali (O19)
Primnoeides sertularoides (SW3181)
Primnoeides sertularoides (SW3149)
Primnoeides sertularoides (SW3165)
Primnoeides sertularoides (SW3202)
Primnoeides sp. n. A [sensu T&R 2015] (SW3564)
Primnoeides sertularoides (SW3174)
Primnoeides sp. n. A [sensu T&R 2015] (SW3291)
Primnoeides sp. n. A [sensu T&R 2015] (SW3297)
Onogorgia nodosa (USNM82940)
Onogorgia nodosa (USNM1184513)
Onogorgia nodosa (AE010)
Onogorgia nodosa (N29)
Onogorgia nodosa (N28)
Onogorgia nodosa (N30)
Fannyella (Scyphogorgia) abies (N60495)
Fannyella (Fannyella) rossi (AR019)
Fannyella (Fannyella) rossi (AR048)
Fannyella (Fannyella) rossi (AR095)
Fannyella (Fannyella) rossi (AR102)
Fannyella (Fannyella) rossi (AR167)
Fannyella (Fannyella) rossi (N37224)
Fannyella (Cyathogorgia) spinosa (N35534)
Fannyella (Cyathogorgia) spinosa (AR085)
Fannyella (Cyathogorgia) spinosa (AR123)
Fannyella (Cyathogorgia) spinosa (AR173)
Fannyella (Cyathogorgia) spinosa (AR013)
Ainigmaptilon c.f. edisto (N01)
Ainigmaptilon edisto (AR105)
Callozostron mirabile (N77603)
Callozostron acanthodes (N78759)
Callozostron acanthodes (AR185)
Callozostron pinnatum (N82682)
Plumarella (Plumarella) laxiramosa (USNM1019406)
Plumarella (Plumarella) longispina (USNM1117110)
Plumarella (Dicholaphis) aleutiana (USNM1134746)
Plumarella c.f. delicatissima (N05)
Plumarella (Plumarella) delicatissima (O24)
Tokoprymno sp. n. [sensu T&R 2015] (PRIM10)
Tokoprymno sp. n [sensu T&R 2015] (PRIM09)
Mirostenella sp. n [sensu T&R 2015] (AP12)
Mirostenella articulata (AC35)
Mirostenella articulata (AE002)
Mirostenella articulata (AE093)
Mirostenella articulata (AE112)
Thouarella c.f. laxa (AR232) Group C
Thouarella laxa (AR194) Group C
Thouarella laxa (AR200) Group C
Thouarella variabilis (AE059) Group A
Thouarella c.f. brucei (T125) Group B
Thouarella c.f. variabilis (AR096) Group A
Thouarella variabilis (AR134) Group A
Thouarella variabilis (AR169) Group A
Thouarella pendulina (AR135) Group A
Thouarella chilensis (N09) Group A
Thouarella variabilis (AR027) Group A
Thouarella cf variabilis (A06) Group A
Thouarella sp. n. A [sensu T&R 2015] (AC28)
Thouarella variabilis (F6) Group A
Thouarella antarctica (AR018) Group A
Plumarella (Plumarella) diadema (Z051)
Plumarella (Plumarella) diadema (AE068)
Plumarella (Plumarella) diadema (S060)
Plumarella (Plumarella) diadema (B34)
Plumarella (Plumarella) undulata (Z046)
Plumarella (Plumarella) undulata (O28)
Plumarella (Plumarella) undulata (B48)
Plumarella (Plumarella) undulata (S093)
Thouarella viridis (AC23) Group A
Thouarella viridis (AE019) Group A
Thouarella viridis (H13) Group A
Thouarella chilensis (R54) Group A
Thouarella antarctica (AE064) Group A
Thouarella chilensis (AE026) Group A
Thouarella antarctica (S170) Group A
Thouarella crenelata (S125) Group A
Thouarella crenelata (W54) Group A
Thouarella crenelata (AE057) Group A
Thouarella crenelata (AR091) Group A
Thouarella cf antarctica (AR175) Group A
1A
1B1
1B
1B2
1C
2
2A
3
3A
4
5
5A
5A1
5A2
5B
5B1
5B2
5B3
ML dichotomous planar = 0.98
ML unbranched = 0.76
ML unbranched = 0.98
ML unbranched = 0.73
ML dichotomous lyriform = 0.79
ML dichotomous bushy = 0.96
ML unbranched = 0.79
Character 1: Colony shape
Parsimony ancestral state reconstruction
unbranched
dichotomous planar
dichotomous (lyriform)
dichotomous (bushy)
dichotomous (sparse)
opposite pinnate
alternate pinnate
bottlebrush
branching from basal bolus
Relative likelihoods for ancestral states discussed in text are shown. For all likelihoods see Additional File 5.

Figure S1a

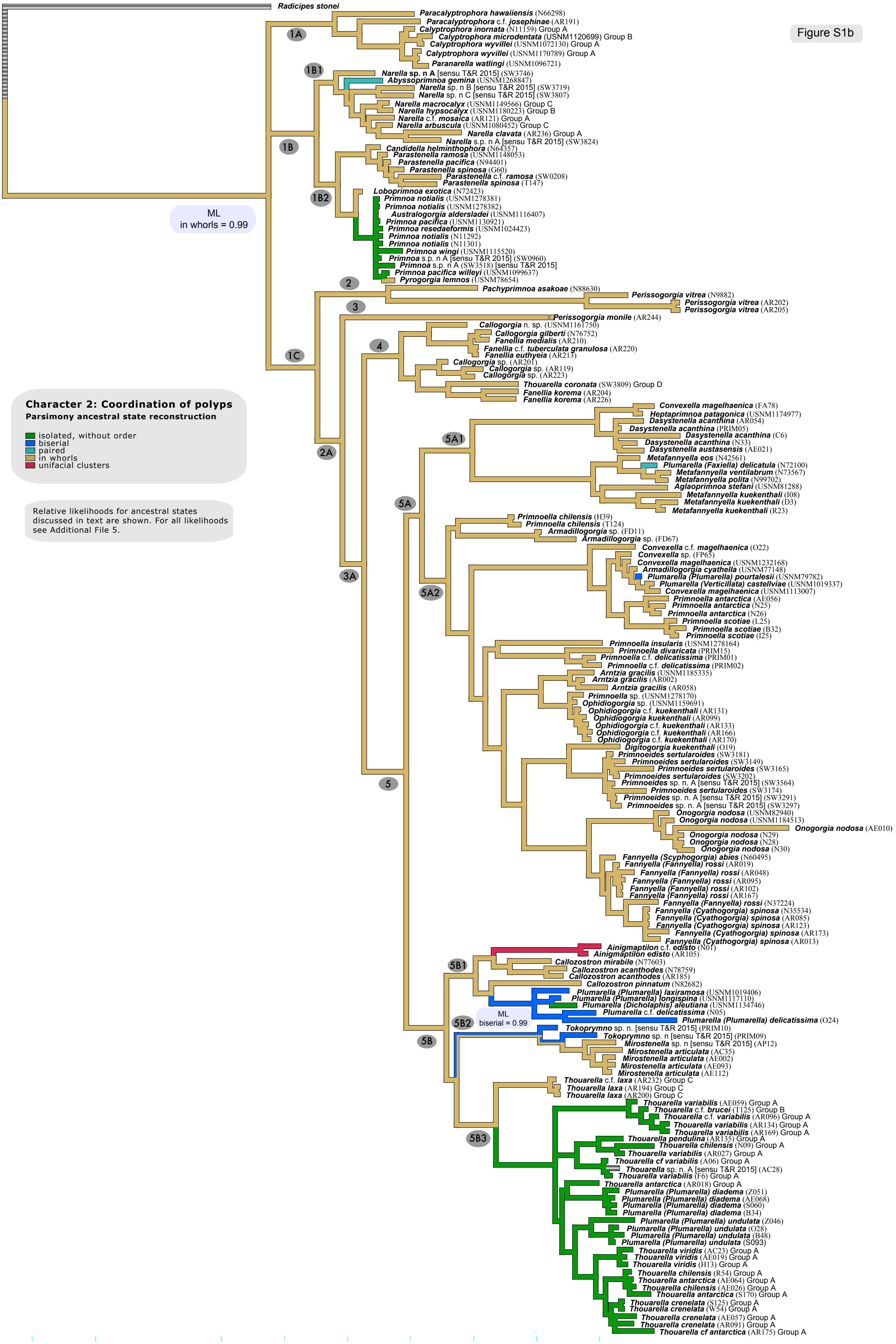

Radicipes stonei
Paracalyptrophora hawaiiensis (N66298)
Paracalyptrophora c.f. josephinae (AR191)
Calyptrophora inornata (N11159) Group A
Calyptrophora microdentata (USNM1120699) Group B
Calyptrophora wyvillei (USNM1072130) Group A
Calyptrophora wyvillei (USNM1170789) Group A
Paranarella watlingi (USNM1096721)
1A
1B1
Narella sp. n A [sensu T&R 2015] (SW3746)
Abyssoprimnoa gemina (USNM1268847)
Narella sp. n B [sensu T&R 2015] (SW3719)
Narella sp. n C [sensu T&R 2015] (SW3807)
Narella macrocalyx (USNM1149566) Group C
Narella hypsocalyx (USNM1180223) Group B
Narella c.f. mosaica (AR121) Group A
Narella arbuscula (USNM1080452) Group C
Narella clavata (AR236) Group A
Narella s.p. n A [sensu T&R 2015] (SW3824)
1B
Candidella helminthophora (N64357)
Parastenella ramosa (USNM1148053)
Parastenella pacifica (N94401)
Parastenella spinosa (G60)
Parastenella c.f. ramosa (SW0208)
Parastenella spinosa (T147)
1B2
Loboprimnoa exotica (N72423)
Primnoa notialis (USNM1278381)
Primnoa notialis (USNM1278382)
Australogorgia aldersladei (USNM1116407)
Primnoa pacifica (USNM1130921)
Primnoa resedaeformis (USNM1024423)
Primnoa notialis (N11292)
Primnoa notialis (N11301)
Primnoa wingi (USNM1115520)
Primnoa s.p. n A [sensu T&R 2015] (SW0960)
Primnoa s.p. n A (SW3518) [sensu T&R 2015]
Primnoa pacifica willeyi (USNM1099637)
Pyrogorgia lemnos (USNM78654)
ML
in whorls = 0.99
2
Pachyprimnoa asakoae (N88630)
Perissogorgia vitrea (N9882)
Perissogorgia vitrea (AR202)
Perissogorgia vitrea (AR205)
3
Perissogorgia monile (AR244)
Callogorgia n. sp. (USNM1161750)
Callogorgia gilberti (N76752)
Fanellia medialis (AR210)
4
Fanellia c.f. tuberculata granulosa (AR220)
1C
Fanellia euthyeia (AR213)
Callogorgia sp. (AR201)
Callogorgia sp. (AR119)
Callogorgia sp. (AR223)
Thouarella coronata (SW3809) Group D
Fanellia korema (AR204)
Fanellia korema (AR226)
Character 2: Coordination of polyps
Parsimony ancestral state reconstruction
isolated, without order
biserial
paired
in whorls
unifacial clusters
Relative likelihoods for ancestral states discussed in text are shown. For all likelihoods see Additional File 5.
Convexella magelhaenica (FA78)
Heptaprimnoa patagonica (USNM1174977)
Dasystenella acanthina (AR054)
Dasystenella acanthina (PRIM05)
Dasystenella acanthina (C6)
Dasystenella acanthina (N33)
Dasystenella austasensis (AE021)
5A1
2A
Metafannyella eos (N42561)
Plumarella (Faxiella) delicatula (N72100)
Metafannyella ventilabrum (N73567)
Metafannyella polita (N99702)
Aglaoprimnoa stefani (USNM81288)
Metafannyella kuekenthali (I08)
Metafannyella kuekenthali (D3)
Metafannyella kuekenthali (R23)
5A
Primnoella chilensis (H39)
Primnoella chilensis (T124)
Armadillogorgia sp. (FD11)
Armadillogorgia sp. (FD67)
Convexella c.f. magelhaenica (O22)
Convexella sp. (FP65)
Convexella magelhaenica (USNM1232168)
Armadillogorgia cyathella (USNM77148)
Plumarella (Plumarella) pourtalesii (USNM79782)
Plumarella (Verticillata) castellviae (USNM1019337)
Convexella magelhaenica (USNM1113007)
3A
5A2
Primnoella antarctica (AE056)
Primnoella antarctica (N25)
Primnoella antarctica (N26)
Primnoella scotiae (L25)
Primnoella scotiae (B32)
Primnoella scotiae (I25)
Primnoella insularis (USNM1278164)
Primnoella divaricata (PRIM15)
Primnoella c.f. delicatissima (PRIM01)
Primnoella c.f. delicatissima (PRIM02)
Arntzia gracilis (USNM1185335)
Arntzia gracilis (AR002)
Arntzia gracilis (AR058)
Primnoella sp. (USNM1278170)
Ophidiogorgia sp. (USNM1159691)
Ophidiogorgia c.f. kuekenthali (AR131)
Ophidiogorgia kuekenthali (AR099)
Ophidiogorgia c.f. kuekenthali (AR133)
Ophidiogorgia c.f. kuekenthali (AR166)
Ophidiogorgia c.f. kuekenthali (AR170)
Digitogorgia kuekenthali (O19)
Primnoeides sertularoides (SW3181)
Primnoeides sertularoides (SW3149)
Primnoeides sertularoides (SW3165)
Primnoeides sertularoides (SW3202)
5
Primnoeides sp. n. A [sensu T&R 2015] (SW3564)
Primnoeides sertularoides (SW3174)
Primnoeides sp. n. A [sensu T&R 2015] (SW3291)
Primnoeides sp. n. A [sensu T&R 2015] (SW3297)
Onogorgia nodosa (USNM82940)
Onogorgia nodosa (USNM1184513)
Onogorgia nodosa (AE010)
Onogorgia nodosa (N29)
Onogorgia nodosa (N28)
Onogorgia nodosa (N30)
Fannyella (Scyphogorgia) abies (N60495)
Fannyella (Fannyella) rossi (AR019)
Fannyella (Fannyella) rossi (AR048)
Fannyella (Fannyella) rossi (AR095)
Fannyella (Fannyella) rossi (AR102)
Fannyella (Fannyella) rossi (AR167)
Fannyella (Fannyella) rossi (N37224)
Fannyella (Cyathogorgia) spinosa (N35534)
Fannyella (Cyathogorgia) spinosa (AR085)
Fannyella (Cyathogorgia) spinosa (AR123)
Fannyella (Cyathogorgia) spinosa (AR173)
Fannyella (Cyathogorgia) spinosa (AR013)
Ainigmaptilon c.f. edisto (N01)
Ainigmaptilon edisto (AR105)
Callozostron mirabile (N77603)
5B1
Callozostron acanthodes (N78759)
Callozostron acanthodes (AR185)
Callozostron pinnatum (N82682)
Plumarella (Plumarella) laxiramosa (USNM1019406)
Plumarella (Plumarella) longispina (USNM1117110)
Plumarella (Dicholaphis) aleutiana (USNM1134746)
Plumarella c.f. delicatissima (N05)
ML
biserial = 0.99
Plumarella (Plumarella) delicatissima (O24)
5B2
Tokoprymno sp. n. [sensu T&R 2015] (PRIM10)
Tokoprymno sp. n [sensu T&R 2015] (PRIM09)
Mirostenella sp. n [sensu T&R 2015] (AP12)
5B
Mirostenella articulata (AC35)
Mirostenella articulata (AE002)
Mirostenella articulata (AE093)
Mirostenella articulata (AE112)
Thouarella c.f. laxa (AR232) Group C
Thouarella laxa (AR194) Group C
Thouarella laxa (AR200) Group C
Thouarella variabilis (AE059) Group A
Thouarella c.f. brucei (T125) Group B
Thouarella c.f. variabilis (AR096) Group A
Thouarella variabilis (AR134) Group A
Thouarella variabilis (AR169) Group A
5B3
Thouarella pendulina (AR135) Group A
Thouarella chilensis (N09) Group A
Thouarella variabilis (AR027) Group A
Thouarella cf variabilis (A06) Group A
Thouarella sp. n. A [sensu T&R 2015] (AC28)
Thouarella variabilis (F6) Group A
Thouarella antarctica (AR018) Group A
Plumarella (Plumarella) diadema (Z051)
Plumarella (Plumarella) diadema (AE068)
Plumarella (Plumarella) diadema (S060)
Plumarella (Plumarella) diadema (B34)
Plumarella (Plumarella) undulata (Z046)
Plumarella (Plumarella) undulata (O28)
Plumarella (Plumarella) undulata (B48)
Plumarella (Plumarella) undulata (S093)
Thouarella viridis (AC23) Group A
Thouarella viridis (AE019) Group A
Thouarella viridis (H13) Group A
Thouarella chilensis (R54) Group A
Thouarella antarctica (AE064) Group A
Thouarella chilensis (AE026) Group A
Thouarella antarctica (S170) Group A
Thouarella crenelata (S125) Group A
Thouarella crenelata (W54) Group A
Thouarella crenelata (AE057) Group A
Thouarella crenelata (AR091) Group A
Thouarella cf antarctica (AR175) Group A

Figure S1b

Figure S1c

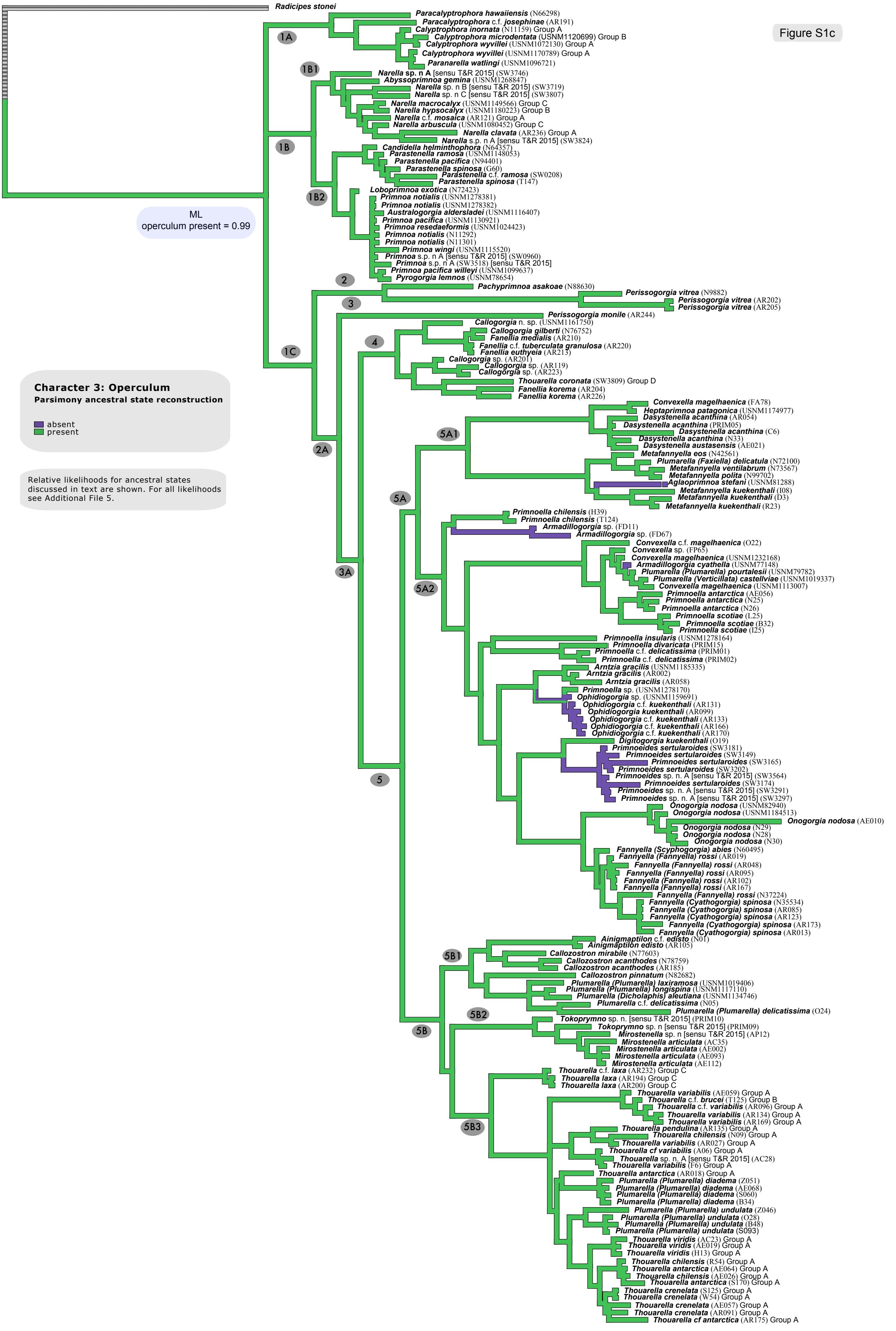
Radicipes stonei
Paracalyptrophora hawaiiensis (N66298)
Paracalyptrophora c.f. josephinae (AR191)
Calyptrophora inornata (N11159) Group A
Calyptrophora microdentata (USNM1120699) Group B
Calyptrophora wyvillei (USNM1072130) Group A
Calyptrophora wyvillei (USNM1170789) Group A
Paranarella watlingi (USNM1096721)
1A
1B1
Narella sp. n A [sensu T&R 2015] (SW3746)
Abyssoprimnoa gemina (USNM1268847)
Narella sp. n B [sensu T&R 2015] (SW3719)
Narella sp. n C [sensu T&R 2015] (SW3807)
Narella macrocalyx (USNM1149566) Group C
Narella hypsocalyx (USNM1180223) Group B
Narella c.f. mosaica (AR121) Group A
Narella arbuscula (USNM1080452) Group C
Narella clavata (AR236) Group A
Narella s.p. n A [sensu T&R 2015] (SW3824)
1B
Candidella helminthophora (N64357)
Parastenella ramosa (USNM1148053)
Parastenella pacifica (N94401)
Parastenella spinosa (G60)
Parastenella c.f. ramosa (SW0208)
Parastenella spinosa (T147)
1B2
Loboprimnoa exotica (N72423)
Primnoa notialis (USNM1278381)
Primnoa notialis (USNM1278382)
Australogorgia aldersladei (USNM1116407)
Primnoa pacifica (USNM1130921)
Primnoa resedaeformis (USNM1024423)
Primnoa notialis (N11292)
Primnoa notialis (N11301)
Primnoa wingi (USNM1115520)
Primnoa s.p. n A [sensu T&R 2015] (SW0960)
Primnoa s.p. n A (SW3518) [sensu T&R 2015]
Primnoa pacifica willeyi (USNM1099637)
Pyrogorgia lemnos (USNM78654)
ML
operculum present = 0.99
2
Pachyprimnoa asakoae (N88630)
Perissogorgia vitrea (N9882)
3
Perissogorgia vitrea (AR202)
Perissogorgia vitrea (AR205)
Perissogorgia monile (AR244)
Callogorgia n. sp. (USNM1161750)
4
Callogorgia gilberti (N76752)
Fanellia medialis (AR210)
Fanellia c.f. tuberculata granulosa (AR220)
Fanellia euthyeia (AR213)
1C
Callogorgia sp. (AR201)
Callogorgia sp. (AR119)
Callogorgia sp. (AR223)
Thouarella coronata (SW3809) Group D
Fanellia korema (AR204)
Fanellia korema (AR226)
Character 3: Operculum
Parsimony ancestral state reconstruction
absent
present
Convexella magelhaenica (FA78)
Heptaprimnoa patagonica (USNM1174977)
Dasystenella acanthina (AR054)
Dasystenella acanthina (PRIM05)
Dasystenella acanthina (C6)
Dasystenella acanthina (N33)
Dasystenella austasensis (AE021)
5A1
2A
Metafannyella eos (N42561)
Plumarella (Faxiella) delicatula (N72100)
Metafannyella ventilabrum (N73567)
Metafannyella polita (N99702)
Aglaoprimnoa stefani (USNM81288)
Metafannyella kuekenthali (I08)
Metafannyella kuekenthali (D3)
Metafannyella kuekenthali (R23)
Relative likelihoods for ancestral states discussed in text are shown. For all likelihoods see Additional File 5.
5A
Primnoella chilensis (H39)
Primnoella chilensis (T124)
Armadillogorgia sp. (FD11)
Armadillogorgia sp. (FD67)
Convexella c.f. magelhaenica (O22)
Convexella sp. (FP65)
Convexella magelhaenica (USNM1232168)
Armadillogorgia cyathella (USNM77148)
Plumarella (Plumarella) pourtalesii (USNM79782)
Plumarella (Verticillata) castellviae (USNM1019337)
Convexella magelhaenica (USNM1113007)
Primnoella antarctica (AE056)
Primnoella antarctica (N25)
Primnoella antarctica (N26)
Primnoella scotiae (L25)
Primnoella scotiae (B32)
Primnoella scotiae (I25)
3A
5A2
Primnoella insularis (USNM1278164)
Primnoella divaricata (PRIM15)
Primnoella c.f. delicatissima (PRIM01)
Primnoella c.f. delicatissima (PRIM02)
Arntzia gracilis (USNM1185335)
Arntzia gracilis (AR002)
Arntzia gracilis (AR058)
Primnoella sp. (USNM1278170)
Ophidiogorgia sp. (USNM1159691)
Ophidiogorgia c.f. kuekenthali (AR131)
Ophidiogorgia kuekenthali (AR099)
Ophidiogorgia c.f. kuekenthali (AR133)
Ophidiogorgia kuekenthali (AR166)
Ophidiogorgia c.f. kuekenthali (AR170)
Digitogorgia kuekenthali (O19)
Primnoeides sertularoides (SW3181)
Primnoeides sertularoides (SW3149)
Primnoeides sertularoides (SW3165)
Primnoeides sertularoides (SW3202)
Primnoeides sp. n. A [sensu T&R 2015] (SW3564)
Primnoeides sertularoides (SW3174)
Primnoeides sp. n. A [sensu T&R 2015] (SW3291)
Primnoeides sp. n. A [sensu T&R 2015] (SW3297)
5
Onogorgia nodosa (USNM82940)
Onogorgia nodosa (USNM1184513)
Onogorgia nodosa (AE010)
Onogorgia nodosa (N29)
Onogorgia nodosa (N28)
Onogorgia nodosa (N30)
Fannyella (Scyphogorgia) abies (N60495)
Fannyella (Fannyella) rossi (AR019)
Fannyella (Fannyella) rossi (AR048)
Fannyella (Fannyella) rossi (AR095)
Fannyella (Fannyella) rossi (AR102)
Fannyella (Fannyella) rossi (AR167)
Fannyella (Fannyella) rossi (N37224)
Fannyella (Cyathogorgia) spinosa (N35534)
Fannyella (Cyathogorgia) spinosa (AR085)
Fannyella (Cyathogorgia) spinosa (AR123)
Fannyella (Cyathogorgia) spinosa (AR173)
Fannyella (Cyathogorgia) spinosa (AR013)
Ainigmaptilon c.f. edisto (N01)
Ainigmaptilon edisto (AR105)
5B1
Callozostron mirabile (N77603)
Callozostron acanthodes (N78759)
Callozostron acanthodes (AR185)
Callozostron pinnatum (N82682)
Plumarella (Plumarella) laxiramosa (USNM1019406)
Plumarella (Plumarella) longispina (USNM1117110)
Plumarella (Dicholaphis) aleutiana (USNM1134746)
Plumarella c.f. delicatissima (N05)
Plumarella (Plumarella) delicatissima (O24)
5B2
Tokoprymno sp. n. [sensu T&R 2015] (PRIM10)
5B
Tokoprymno sp. n [sensu T&R 2015] (PRIM09)
Mirostenella sp. n [sensu T&R 2015] (AP12)
Mirostenella articulata (AC35)
Mirostenella articulata (AE002)
Mirostenella articulata (AE093)
Mirostenella articulata (AE112)
Thouarella c.f. laxa (AR232) Group C
Thouarella laxa (AR194) Group C
Thouarella laxa (AR200) Group C
Thouarella variabilis (AE059) Group A
Thouarella c.f. brucei (T125) Group B
Thouarella c.f. variabilis (AR096) Group A
Thouarella variabilis (AR134) Group A
Thouarella variabilis (AR169) Group A
5B3
Thouarella pendulina (AR135) Group A
Thouarella chilensis (N09) Group A
Thouarella variabilis (AR027) Group A
Thouarella cf variabilis (A06) Group A
Thouarella sp. n. A [sensu T&R 2015] (AC28)
Thouarella variabilis (F6) Group A
Thouarella antarctica (AR018) Group A
Plumarella (Plumarella) diadema (Z051)
Plumarella (Plumarella) diadema (AE068)
Plumarella (Plumarella) diadema (S060)
Plumarella (Plumarella) diadema (B34)
Plumarella (Plumarella) undulata (Z046)
Plumarella (Plumarella) undulata (O28)
Plumarella (Plumarella) undulata (B48)
Plumarella (Plumarella) undulata (S093)
Thouarella viridis (AC23) Group A
Thouarella viridis (AE019) Group A
Thouarella viridis (H13) Group A
Thouarella chilensis (R54) Group A
Thouarella antarctica (AE064) Group A
Thouarella chilensis (AE026) Group A
Thouarella antarctica (S170) Group A
Thouarella crenelata (S125) Group A
Thouarella crenelata (W54) Group A
Thouarella crenelata (AE057) Group A
Thouarella crenelata (AR091) Group A
Thouarella cf antarctica (AR175) Group A

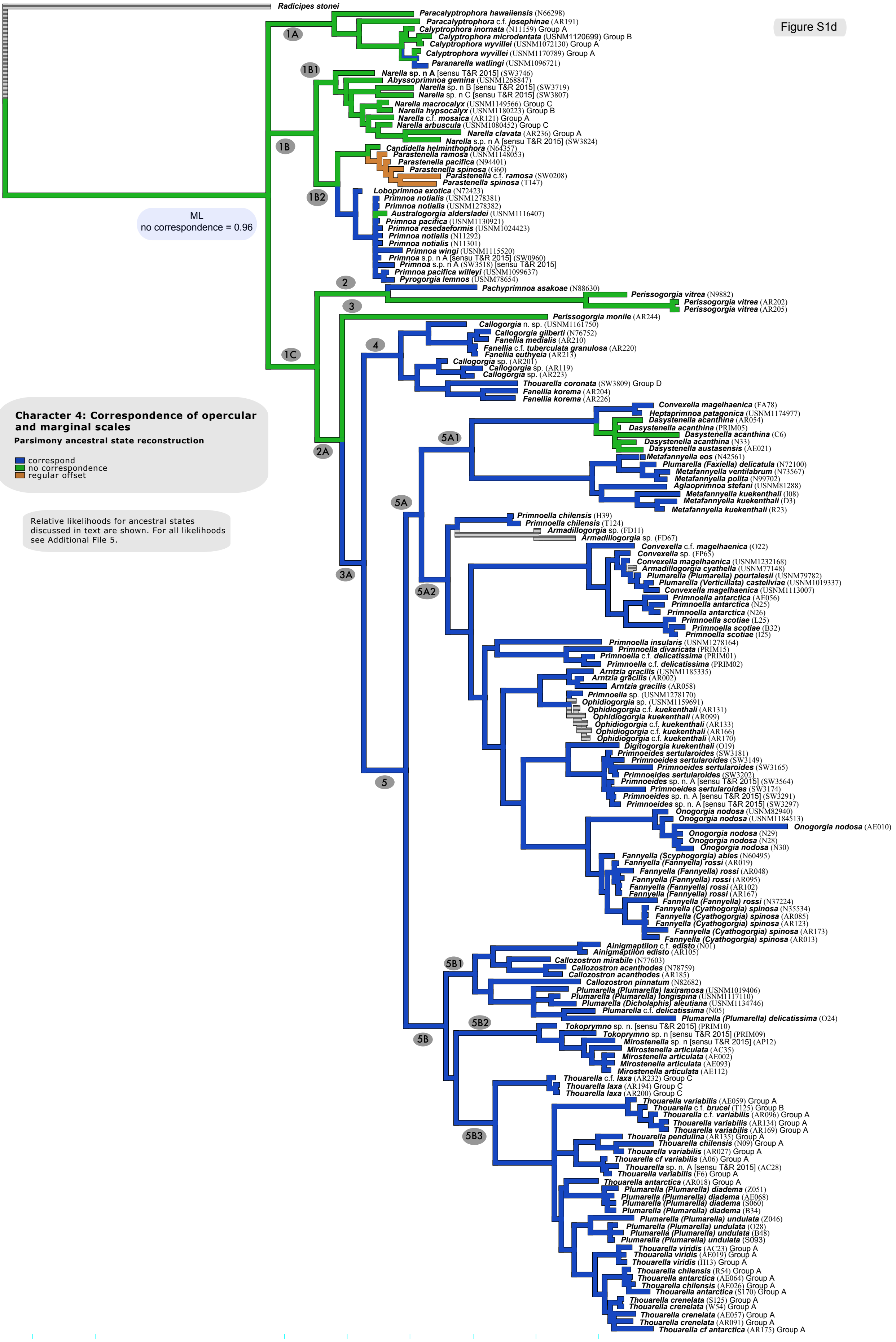

Figure S1d
Radicipes stonei
Paracalyptrophora hawaiiensis (N66298)
Paracalyptrophora c.f. josephinae (AR191)
Calyptrophora inornata (N11159) Group A
Calyptrophora microdentata (USNM1120699) Group B
Calyptrophora wyvillei (USNM1072130) Group A
Calyptrophora wyvillei (USNM1170789) Group A
Paranarella watlingi (USNM1096721)
Narella sp. n A [sensu T&R 2015] (SW3746)
Abyssoprimnoa gemina (USNM1268847)
Narella sp. n B [sensu T&R 2015] (SW3719)
Narella sp. n C [sensu T&R 2015] (SW3807)
Narella macrocalyx (USNM1149566) Group C
Narella hypsocalyx (USNM1180223) Group B
Narella c.f. mosaica (AR121) Group A
Narella arbuscula (USNM1080452) Group C
Narella clavata (AR236) Group A
Narella s.p. n A [sensu T&R 2015] (SW3824)
Candidella helminthophora (N64357)
Parastenella ramosa (USNM1148053)
Parastenella pacifica (N94401)
Parastenella spinosa (G60)
Parastenella c.f. ramosa (SW0208)
Parastenella spinosa (T147)
Loboprimnoa exotica (N72423)
Primnoa notialis (USNM1278381)
Primnoa notialis (USNM1278382)
Australogorgia aldersladei (USNM1116407)
Primnoa pacifica (USNM1130921)
Primnoa resedaeformis (USNM1024423)
Primnoa notialis (N11292)
Primnoa notialis (N11301)
Primnoa wingi (USNM1115520)
Primnoa s.p. n A [sensu T&R 2015] (SW0960)
Primnoa s.p. n A (SW3518) [sensu T&R 2015]
Primnoa pacifica willeyi (USNM1099637)
Pyrogorgia lemnos (USNM78654)
Pachyprimnoa asakoae (N88630)
Perissogorgia vitrea (N9882)
Perissogorgia vitrea (AR202)
Perissogorgia vitrea (AR205)
Perissogorgia monile (AR244)
Callogorgia n. sp. (USNM1161750)
Callogorgia gilberti (N76752)
Fanellia medialis (AR210)
Fanellia c.f. tuberculata granulosa (AR220)
Fanellia euthyeia (AR213)
Callogorgia sp. (AR201)
Callogorgia sp. (AR119)
Callogorgia sp. (AR223)
Thouarella coronata (SW3809) Group D
Fanellia korema (AR204)
Fanellia korema (AR226)
Convexella magelhaenica (FA78)
Heptaprimnoa patagonica (USNM1174977)
Dasystenella acanthina (AR054)
Dasystenella acanthina (PRIM05)
Dasystenella acanthina (C6)
Dasystenella acanthina (N33)
Dasystenella austasensis (AE021)
Metafannyella eos (N42561)
Plumarella (Faxiella) delicatula (N72100)
Metafannyella ventilabrum (N73567)
Metafannyella polita (N99702)
Aglaoprimnoa stefani (USNM81288)
Metafannyella kuekenthali (I08)
Metafannyella kuekenthali (D3)
Metafannyella kuekenthali (R23)
Primnoella chilensis (H39)
Primnoella chilensis (T124)
Armadillogorgia sp. (FD11)
Armadillogorgia sp. (FD67)
Convexella c.f. magelhaenica (O22)
Convexella sp. (FP65)
Convexella magelhaenica (USNM1232168)
Armadillogorgia cyathella (USNM77148)
Plumarella (Plumarella) pourtalesii (USNM79782)
Plumarella (Verticillata) castellviae (USNM1019337)
Convexella magelhaenica (USNM1113007)
Primnoella antarctica (AE056)
Primnoella antarctica (N25)
Primnoella antarctica (N26)
Primnoella scotiae (L25)
Primnoella scotiae (B32)
Primnoella scotiae (I25)
Primnoella insularis (USNM1278164)
Primnoella divaricata (PRIM15)
Primnoella c.f. delicatissima (PRIM01)
Primnoella c.f. delicatissima (PRIM02)
Arntzia gracilis (USNM1185335)
Arntzia gracilis (AR002)
Arntzia gracilis (AR058)
Primnoella sp. (USNM1278170)
Ophidiogorgia sp. (USNM1159691)
Ophidiogorgia c.f. kuekenthali (AR131)
Ophidiogorgia kuekenthali (AR099)
Ophidiogorgia c.f. kuekenthali (AR133)
Ophidiogorgia c.f. kuekenthali (AR166)
Ophidiogorgia c.f. kuekenthali (AR170)
Digitogorgia kuekenthali (O19)
Primnoeides sertularoides (SW3181)
Primnoeides sertularoides (SW3149)
Primnoeides sertularoides (SW3165)
Primnoeides sertularoides (SW3202)
Primnoeides sp. n. A [sensu T&R 2015] (SW3564)
Primnoeides sertularoides (SW3174)
Primnoeides sp. n. A [sensu T&R 2015] (SW3291)
Primnoeides sp. n. A [sensu T&R 2015] (SW3297)
Onogorgia nodosa (USNM82940)
Onogorgia nodosa (USNM1184513)
Onogorgia nodosa (AE010)
Onogorgia nodosa (N29)
Onogorgia nodosa (N28)
Onogorgia nodosa (N30)
Fannyella (Scyphogorgia) abies (N60495)
Fannyella (Fannyella) rossi (AR019)
Fannyella (Fannyella) rossi (AR048)
Fannyella (Fannyella) rossi (AR095)
Fannyella (Fannyella) rossi (AR102)
Fannyella (Fannyella) rossi (AR167)
Fannyella (Fannyella) rossi (N37224)
Fannyella (Cyathogorgia) spinosa (N35534)
Fannyella (Cyathogorgia) spinosa (AR085)
Fannyella (Cyathogorgia) spinosa (AR123)
Fannyella (Cyathogorgia) spinosa (AR173)
Fannyella (Cyathogorgia) spinosa (AR013)
Ainigmaptilon c.f. edisto (N01)
Ainigmaptilon edisto (AR105)
Callozostron mirabile (N77603)
Callozostron acanthodes (N78759)
Callozostron acanthodes (AR185)
Callozostron pinnatum (N82682)
Plumarella (Plumarella) laxiramosa (USNM1019406)
Plumarella (Plumarella) longispina (USNM1117110)
Plumarella (Dicholaphis) aleutiana (USNM1134746)
Plumarella c.f. delicatissima (N05)
Plumarella (Plumarella) delicatissima (O24)
Tokoprymno sp. n. [sensu T&R 2015] (PRIM10)
Tokoprymno sp. n [sensu T&R 2015] (PRIM09)
Mirostenella sp. n [sensu T&R 2015] (AP12)
Mirostenella articulata (AC35)
Mirostenella articulata (AE002)
Mirostenella articulata (AE093)
Mirostenella articulata (AE112)
Thouarella c.f. laxa (AR232) Group C
Thouarella laxa (AR194) Group C
Thouarella laxa (AR200) Group C
Thouarella variabilis (AE059) Group A
Thouarella c.f. brucei (T125) Group B
Thouarella c.f. variabilis (AR096) Group A
Thouarella variabilis (AR134) Group A
Thouarella variabilis (AR169) Group A
Thouarella pendulina (AR135) Group A
Thouarella chilensis (N09) Group A
Thouarella variabilis (AR027) Group A
Thouarella cf variabilis (A06) Group A
Thouarella sp. n. A [sensu T&R 2015] (AC28)
Thouarella variabilis (F6) Group A
Thouarella antarctica (AR018) Group A
Plumarella (Plumarella) diadema (Z051)
Plumarella (Plumarella) diadema (AE068)
Plumarella (Plumarella) diadema (S060)
Plumarella (Plumarella) diadema (B34)
Plumarella (Plumarella) undulata (Z046)
Plumarella (Plumarella) undulata (O28)
Plumarella (Plumarella) undulata (B48)
Plumarella (Plumarella) undulata (S093)
Thouarella viridis (AC23) Group A
Thouarella viridis (AE019) Group A
Thouarella viridis (H13) Group A
Thouarella chilensis (R54) Group A
Thouarella antarctica (AE064) Group A
Thouarella chilensis (AE026) Group A
Thouarella antarctica (S170) Group A
Thouarella crenelata (S125) Group A
Thouarella crenelata (W54) Group A
Thouarella crenelata (AE057) Group A
Thouarella crenelata (AR091) Group A
Thouarella cf antarctica (AR175) Group A
1A
1B1
1B
1B2
1C
2
2A
3
3A
4
5
5A
5A1
5A2
5B
5B1
5B2
5B3
ML
no correspondence = 0.96
Character 4: Correspondence of opercular and marginal scales
Parsimony ancestral state reconstruction
correspond
no correspondence
regular offset
Relative likelihoods for ancestral states discussed in text are shown. For all likelihoods see Additional File 5.

Figure S1e

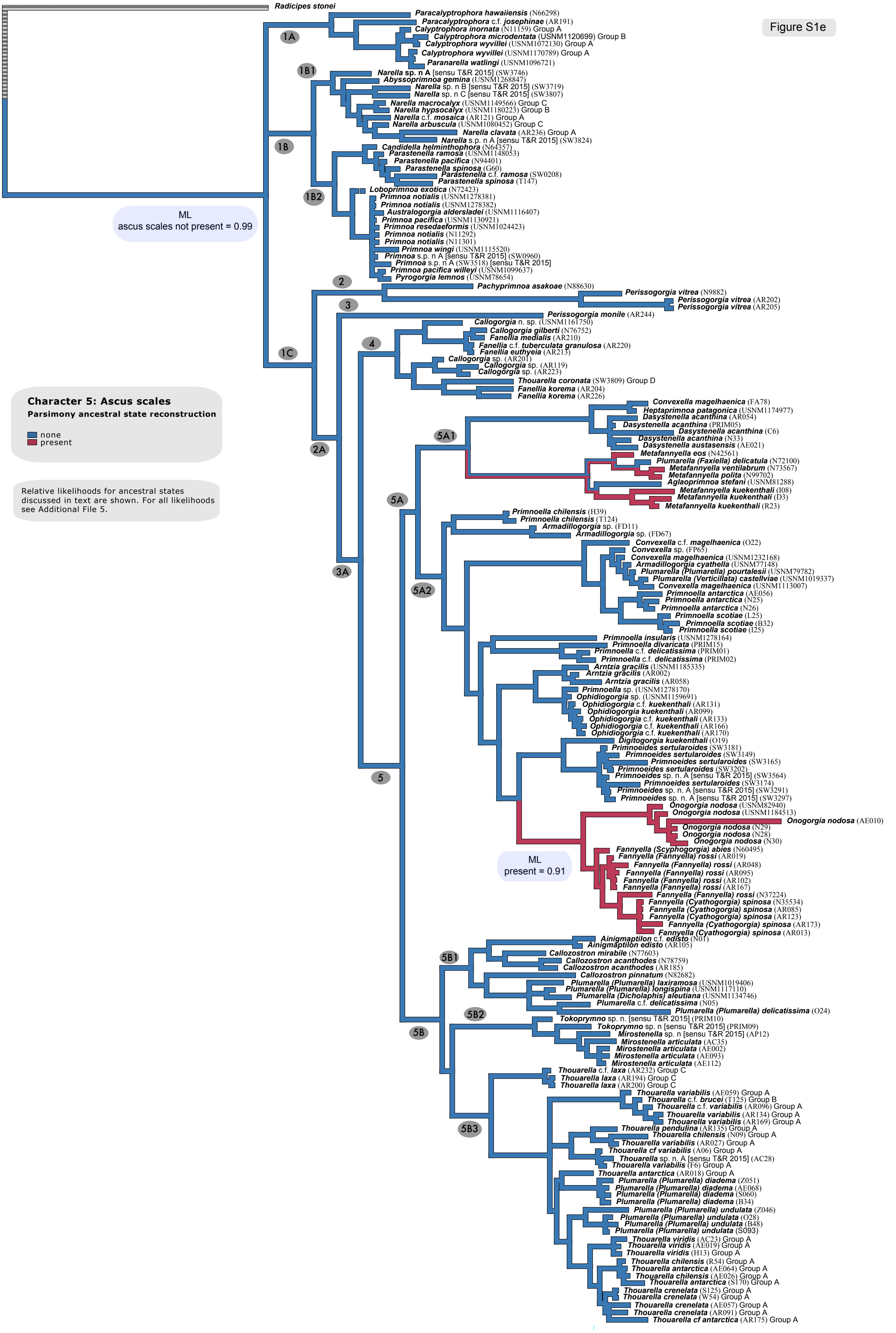

Radicipes stonei
Paracalyptrophora hawaiiensis (N66298)
Paracalyptrophora c.f. josephinae (AR191)
Calyptrophora inornata (N11159) Group A
Calyptrophora microdentata (USNM1120699) Group B
Calyptrophora wyvillei (USNM1072130) Group A
Calyptrophora wyvillei (USNM1170789) Group A
Paranarella watlingi (USNM1096721)
Narella sp. n A [sensu T&R 2015] (SW3746)
Abyssoprimnoa gemina (USNM1268847)
Narella sp. n B [sensu T&R 2015] (SW3719)
Narella sp. n C [sensu T&R 2015] (SW3807)
Narella macrocalyx (USNM1149566) Group C
Narella hypsocalyx (USNM1180223) Group B
Narella c.f. mosaica (AR121) Group A
Narella arbuscula (USNM1080452) Group C
Narella clavata (AR236) Group A
Narella s.p. n A [sensu T&R 2015] (SW3824)
Candidella helminthophora (N64357)
Parastenella ramosa (USNM1148053)
Parastenella pacifica (N94401)
Parastenella spinosa (G60)
Parastenella c.f. ramosa (SW0208)
Parastenella spinosa (T147)
Loboprimnoa exotica (N72423)
Primnoa notialis (USNM1278381)
Primnoa notialis (USNM1278382)
Australogorgia aldersladei (USNM1116407)
Primnoa pacifica (USNM1130921)
Primnoa resedaeformis (USNM1024423)
Primnoa notialis (N11292)
Primnoa notialis (N11301)
Primnoa wingi (USNM1115520)
Primnoa s.p. n A [sensu T&R 2015] (SW0960)
Primnoa s.p. n A (SW3518) [sensu T&R 2015]
Primnoa pacifica willeyi (USNM1099637)
Pyrogorgia lemnos (USNM78654)
Pachyprimnoa asakoae (N88630)
Perissogorgia vitrea (N9882)
Perissogorgia vitrea (AR202)
Perissogorgia vitrea (AR205)
Perissogorgia monile (AR244)
Callogorgia n. sp. (USNM1161750)
Callogorgia gilberti (N76752)
Fanellia medialis (AR210)
Fanellia c.f. tuberculata granulosa (AR220)
Fanellia euthyeia (AR213)
Callogorgia sp. (AR201)
Callogorgia sp. (AR119)
Callogorgia sp. (AR223)
Thouarella coronata (SW3809) Group D
Fanellia korema (AR204)
Fanellia korema (AR226)
Convexella magelhaenica (FA78)
Heptaprimnoa patagonica (USNM1174977)
Dasystenella acanthina (AR054)
Dasystenella acanthina (PRIM05)
Dasystenella acanthina (C6)
Dasystenella acanthina (N33)
Dasystenella austasensis (AE021)
Metafannyella eos (N42561)
Plumarella (Faxiella) delicatula (N72100)
Metafannyella ventilabrum (N73567)
Metafannyella polita (N99702)
Aglaoprimnoa stefani (USNM81288)
Metafannyella kuekenthali (I08)
Metafannyella kuekenthali (D3)
Metafannyella kuekenthali (R23)
Primnoella chilensis (H39)
Primnoella chilensis (T124)
Armadillogorgia sp. (FD11)
Armadillogorgia sp. (FD67)
Convexella c.f. magelhaenica (O22)
Convexella sp. (FP65)
Convexella magelhaenica (USNM1232168)
Armadillogorgia cyathella (USNM77148)
Plumarella (Plumarella) pourtalesii (USNM79782)
Plumarella (Verticillata) castellviae (USNM1019337)
Convexella magelhaenica (USNM1113007)
Primnoella antarctica (AE056)
Primnoella antarctica (N25)
Primnoella antarctica (N26)
Primnoella scotiae (L25)
Primnoella scotiae (B32)
Primnoella scotiae (I25)
Primnoella insularis (USNM1278164)
Primnoella divaricata (PRIM15)
Primnoella c.f. delicatissima (PRIM01)
Primnoella c.f. delicatissima (PRIM02)
Arntzia gracilis (USNM1185335)
Arntzia gracilis (AR002)
Arntzia gracilis (AR058)
Primnoella sp. (USNM1278170)
Ophidiogorgia sp. (USNM1159691)
Ophidiogorgia c.f. kuekenthali (AR131)
Ophidiogorgia kuekenthali (AR099)
Ophidiogorgia c.f. kuekenthali (AR133)
Ophidiogorgia c.f. kuekenthali (AR166)
Ophidiogorgia c.f. kuekenthali (AR170)
Digitogorgia kuekenthali (O19)
Primnoeides sertularoides (SW3181)
Primnoeides sertularoides (SW3149)
Primnoeides sertularoides (SW3165)
Primnoeides sertularoides (SW3202)
Primnoeides sp. n. A [sensu T&R 2015] (SW3564)
Primnoeides sertularoides (SW3174)
Primnoeides sp. n. A [sensu T&R 2015] (SW3291)
Primnoeides sp. n. A [sensu T&R 2015] (SW3297)
Onogorgia nodosa (USNM82940)
Onogorgia nodosa (USNM1184513)
Onogorgia nodosa (AE010)
Onogorgia nodosa (N29)
Onogorgia nodosa (N28)
Onogorgia nodosa (N30)
Fannyella (Scyphogorgia) abies (N60495)
Fannyella (Fannyella) rossi (AR019)
Fannyella (Fannyella) rossi (AR048)
Fannyella (Fannyella) rossi (AR095)
Fannyella (Fannyella) rossi (AR102)
Fannyella (Fannyella) rossi (AR167)
Fannyella (Fannyella) rossi (N37224)
Fannyella (Cyathogorgia) spinosa (N35534)
Fannyella (Cyathogorgia) spinosa (AR085)
Fannyella (Cyathogorgia) spinosa (AR123)
Fannyella (Cyathogorgia) spinosa (AR173)
Fannyella (Cyathogorgia) spinosa (AR013)
Ainigmaptilon c.f. edisto (N01)
Ainigmaptilon edisto (AR105)
Callozostron mirabile (N77603)
Callozostron acanthodes (N78759)
Callozostron acanthodes (AR185)
Callozostron pinnatum (N82682)
Plumarella (Plumarella) laxiramosa (USNM1019406)
Plumarella (Plumarella) longispina (USNM1117110)
Plumarella (Dicholaphis) aleutiana (USNM1134746)
Plumarella c.f. delicatissima (N05)
Plumarella (Plumarella) delicatissima (O24)
Tokoprymno sp. n. [sensu T&R 2015] (PRIM10)
Tokoprymno sp. n [sensu T&R 2015] (PRIM09)
Mirostenella sp. n [sensu T&R 2015] (AP12)
Mirostenella articulata (AC35)
Mirostenella articulata (AE002)
Mirostenella articulata (AE093)
Mirostenella articulata (AE112)
Thouarella c.f. laxa (AR232) Group C
Thouarella laxa (AR194) Group C
Thouarella laxa (AR200) Group C
Thouarella variabilis (AE059) Group A
Thouarella c.f. brucei (T125) Group B
Thouarella c.f. variabilis (AR096) Group A
Thouarella variabilis (AR134) Group A
Thouarella variabilis (AR169) Group A
Thouarella pendulina (AR135) Group A
Thouarella chilensis (N09) Group A
Thouarella variabilis (AR027) Group A
Thouarella cf variabilis (A06) Group A
Thouarella sp. n. A [sensu T&R 2015] (AC28)
Thouarella variabilis (F6) Group A
Thouarella antarctica (AR018) Group A
Plumarella (Plumarella) diadema (Z051)
Plumarella (Plumarella) diadema (AE068)
Plumarella (Plumarella) diadema (S060)
Plumarella (Plumarella) diadema (B34)
Plumarella (Plumarella) undulata (Z046)
Plumarella (Plumarella) undulata (O28)
Plumarella (Plumarella) undulata (B48)
Plumarella (Plumarella) undulata (S093)
Thouarella viridis (AC23) Group A
Thouarella viridis (AE019) Group A
Thouarella viridis (H13) Group A
Thouarella chilensis (R54) Group A
Thouarella antarctica (AE064) Group A
Thouarella chilensis (AE026) Group A
Thouarella antarctica (S170) Group A
Thouarella crenelata (S125) Group A
Thouarella crenelata (W54) Group A
Thouarella crenelata (AE057) Group A
Thouarella crenelata (AR091) Group A
Thouarella cf antarctica (AR175) Group A
1A
1B1
1B
1B2
1C
2
3
4
2A
3A
5A1
5A
5A2
5
5B1
5B2
5B
5B3
ML
ascus scales not present = 0.99
ML
present = 0.91
Character 5: Ascus scales
Parsimony ancestral state reconstruction
none
present
Relative likelihoods for ancestral states discussed in text are shown. For all likelihoods see Additional File 5.

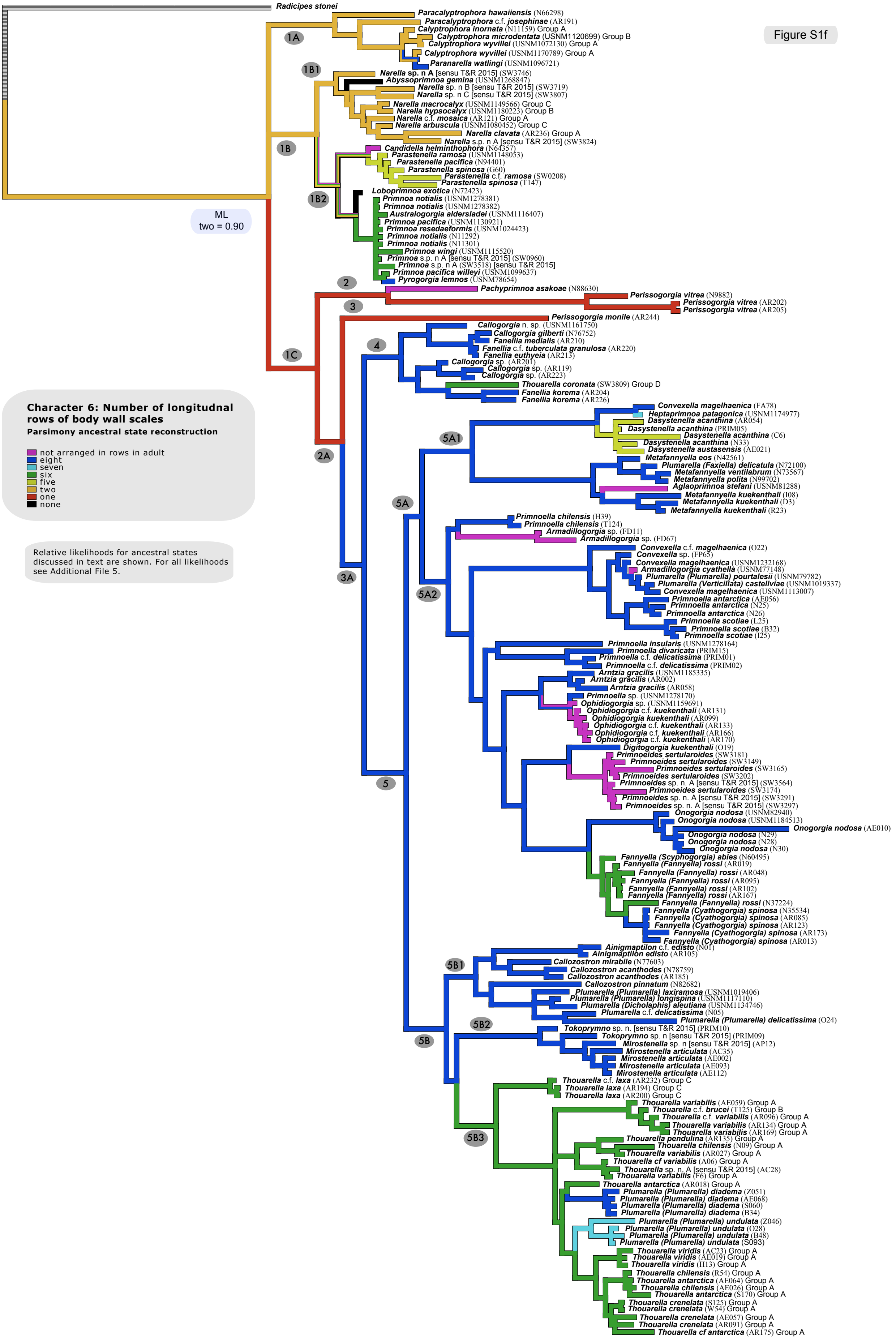

Figure S1f
Radicipes stonei
Paracalyptrophora hawaiiensis (N66298)
Paracalyptrophora c.f. josephinae (AR191)
Calyptrophora inornata (N11159) Group A
Calyptrophora microdentata (USNM1120699) Group B
Calyptrophora wyvillei (USNM1072130) Group A
Calyptrophora wyvillei (USNM1170789) Group A
Paranarella watlingi (USNM1096721)
Narella sp. n A [sensu T&R 2015] (SW3746)
Abyssoprimnoa gemina (USNM1268847)
Narella sp. n B [sensu T&R 2015] (SW3719)
Narella sp. n C [sensu T&R 2015] (SW3807)
Narella macrocalyx (USNM1149566) Group C
Narella hypsocalyx (USNM1180223) Group B
Narella c.f. mosaica (AR121) Group A
Narella arbuscula (USNM1080452) Group C
Narella clavata (AR236) Group A
Narella s.p. n A [sensu T&R 2015] (SW3824)
Candidella helminthophora (N64357)
Parastenella ramosa (USNM1148053)
Parastenella pacifica (N94401)
Parastenella spinosa (G60)
Parastenella c.f. ramosa (SW0208)
Parastenella spinosa (T147)
Loboprimnoa exotica (N72423)
Primnoa notialis (USNM1278381)
Primnoa notialis (USNM1278382)
Australogorgia aldersladei (USNM1116407)
Primnoa pacifica (USNM1130921)
Primnoa resedaeformis (USNM1024423)
Primnoa notialis (N11292)
Primnoa notialis (N11301)
Primnoa wingi (USNM1115520)
Primnoa s.p. n A [sensu T&R 2015] (SW0960)
Primnoa s.p. n A (SW3518) [sensu T&R 2015]
Primnoa pacifica willeyi (USNM1099637)
Pyrogorgia lemnos (USNM78654)
Pachyprimnoa asakoae (N88630)
Perissogorgia vitrea (N9882)
Perissogorgia vitrea (AR202)
Perissogorgia vitrea (AR205)
Perissogorgia monile (AR244)
Callogorgia n. sp. (USNM1161750)
Callogorgia gilberti (N76752)
Fanellia medialis (AR210)
Fanellia c.f. tuberculata granulosa (AR220)
Fanellia euthyeia (AR213)
Callogorgia sp. (AR201)
Callogorgia sp. (AR119)
Callogorgia sp. (AR223)
Thouarella coronata (SW3809) Group D
Fanellia korema (AR204)
Fanellia korema (AR226)
Convexella magelhaenica (FA78)
Heptaprimnoa patagonica (USNM1174977)
Dasystenella acanthina (AR054)
Dasystenella acanthina (PRIM05)
Dasystenella acanthina (C6)
Dasystenella acanthina (N33)
Dasystenella austasensis (AE021)
Metafannyella eos (N42561)
Plumarella (Faxiella) delicatula (N72100)
Metafannyella ventilabrum (N73567)
Metafannyella polita (N99702)
Aglaoprimnoa stefani (USNM81288)
Metafannyella kuekenthali (I08)
Metafannyella kuekenthali (D3)
Metafannyella kuekenthali (R23)
Primnoella chilensis (H39)
Primnoella chilensis (T124)
Armadillogorgia sp. (FD11)
Armadillogorgia sp. (FD67)
Convexella c.f. magelhaenica (O22)
Convexella sp. (FP65)
Convexella magelhaenica (USNM1232168)
Armadillogorgia cyathella (USNM77148)
Plumarella (Plumarella) pourtalesii (USNM79782)
Plumarella (Verticillata) castellviae (USNM1019337)
Convexella magelhaenica (USNM1113007)
Primnoella antarctica (AE056)
Primnoella antarctica (N25)
Primnoella antarctica (N26)
Primnoella scotiae (L25)
Primnoella scotiae (B32)
Primnoella scotiae (I25)
Primnoella insularis (USNM1278164)
Primnoella divaricata (PRIM15)
Primnoella c.f. delicatissima (PRIM01)
Primnoella c.f. delicatissima (PRIM02)
Arntzia gracilis (USNM1185335)
Arntzia gracilis (AR002)
Arntzia gracilis (AR058)
Primnoella sp. (USNM1278170)
Ophidiogorgia sp. (USNM1159691)
Ophidiogorgia c.f. kuekenthali (AR131)
Ophidiogorgia kuekenthali (AR099)
Ophidiogorgia c.f. kuekenthali (AR133)
Ophidiogorgia c.f. kuekenthali (AR166)
Ophidiogorgia c.f. kuekenthali (AR170)
Digitogorgia kuekenthali (O19)
Primnoeides sertularoides (SW3181)
Primnoeides sertularoides (SW3149)
Primnoeides sertularoides (SW3165)
Primnoeides sertularoides (SW3202)
Primnoeides sp. n. A [sensu T&R 2015] (SW3564)
Primnoeides sertularoides (SW3174)
Primnoeides sp. n. A [sensu T&R 2015] (SW3291)
Primnoeides sp. n. A [sensu T&R 2015] (SW3297)
Onogorgia nodosa (USNM82940)
Onogorgia nodosa (USNM1184513)
Onogorgia nodosa (AE010)
Onogorgia nodosa (N29)
Onogorgia nodosa (N28)
Onogorgia nodosa (N30)
Fannyella (Scyphogorgia) abies (N60495)
Fannyella (Fannyella) rossi (AR019)
Fannyella (Fannyella) rossi (AR048)
Fannyella (Fannyella) rossi (AR095)
Fannyella (Fannyella) rossi (AR102)
Fannyella (Fannyella) rossi (AR167)
Fannyella (Fannyella) rossi (N37224)
Fannyella (Cyathogorgia) spinosa (N35534)
Fannyella (Cyathogorgia) spinosa (AR085)
Fannyella (Cyathogorgia) spinosa (AR123)
Fannyella (Cyathogorgia) spinosa (AR173)
Fannyella (Cyathogorgia) spinosa (AR013)
Ainigmaptilon c.f. edisto (N01)
Ainigmaptilon edisto (AR105)
Callozostron mirabile (N77603)
Callozostron acanthodes (N78759)
Callozostron acanthodes (AR185)
Callozostron pinnatum (N82682)
Plumarella (Plumarella) laxiramosa (USNM1019406)
Plumarella (Plumarella) longispina (USNM1117110)
Plumarella (Dicholaphis) aleutiana (USNM1134746)
Plumarella c.f. delicatissima (N05)
Plumarella (Plumarella) delicatissima (O24)
Tokoprymno sp. n. [sensu T&R 2015] (PRIM10)
Tokoprymno sp. n [sensu T&R 2015] (PRIM09)
Mirostenella sp. n [sensu T&R 2015] (AP12)
Mirostenella articulata (AC35)
Mirostenella articulata (AE002)
Mirostenella articulata (AE093)
Mirostenella articulata (AE112)
Thouarella c.f. laxa (AR232) Group C
Thouarella laxa (AR194) Group C
Thouarella laxa (AR200) Group C
Thouarella variabilis (AE059) Group A
Thouarella c.f. brucei (T125) Group B
Thouarella c.f. variabilis (AR096) Group A
Thouarella variabilis (AR134) Group A
Thouarella variabilis (AR169) Group A
Thouarella pendulina (AR135) Group A
Thouarella chilensis (N09) Group A
Thouarella variabilis (AR027) Group A
Thouarella cf variabilis (A06) Group A
Thouarella sp. n. A [sensu T&R 2015] (AC28)
Thouarella variabilis (F6) Group A
Thouarella antarctica (AR018) Group A
Plumarella (Plumarella) diadema (Z051)
Plumarella (Plumarella) diadema (AE068)
Plumarella (Plumarella) diadema (S060)
Plumarella (Plumarella) diadema (B34)
Plumarella (Plumarella) undulata (Z046)
Plumarella (Plumarella) undulata (O28)
Plumarella (Plumarella) undulata (B48)
Plumarella (Plumarella) undulata (S093)
Thouarella viridis (AC23) Group A
Thouarella viridis (AE019) Group A
Thouarella viridis (H13) Group A
Thouarella chilensis (R54) Group A
Thouarella antarctica (AE064) Group A
Thouarella chilensis (AE026) Group A
Thouarella antarctica (S170) Group A
Thouarella crenelata (S125) Group A
Thouarella crenelata (W54) Group A
Thouarella crenelata (AE057) Group A
Thouarella crenelata (AR091) Group A
Thouarella cf antarctica (AR175) Group A
1A
1B1
1B
1B2
2
3
1C
4
2A
3A
5A1
5A
5A2
5
5B1
5B2
5B
5B3
ML
two = 0.90
Character 6: Number of longitudnal rows of body wall scales
Parsimony ancestral state reconstruction
not arranged in rows in adult
eight
seven
six
five
two
one
none
Relative likelihoods for ancestral states discussed in text are shown. For all likelihoods see Additional File 5.

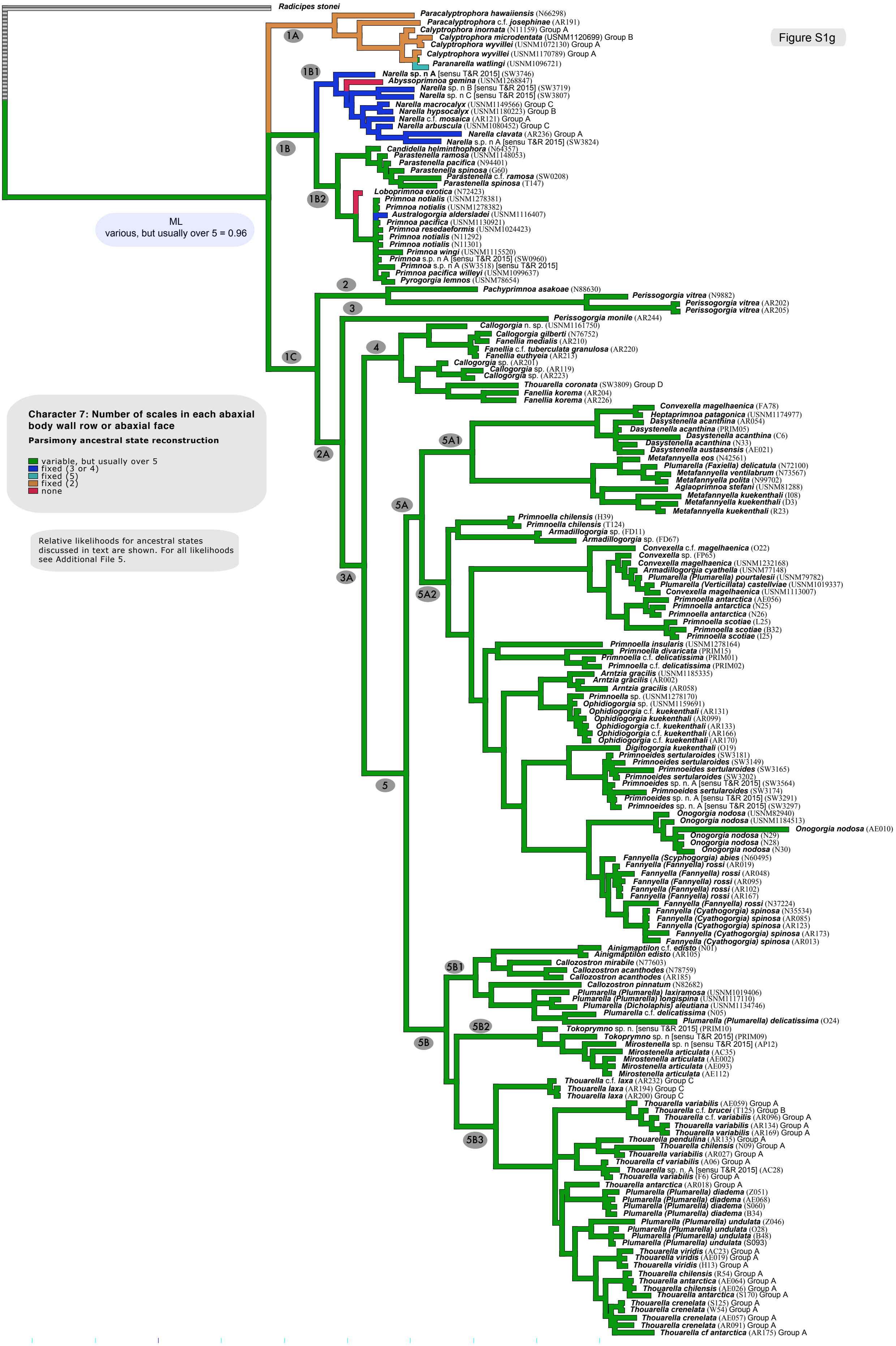
Radicipes stonei
Paracalyptrophora hawaiiensis (N66298)
Paracalyptrophora c.f. josephinae (AR191)
Calyptrophora inornata (N11159) Group A
Calyptrophora microdentata (USNM1120699) Group B
Calyptrophora wyvillei (USNM1072130) Group A
Calyptrophora wyvillei (USNM1170789) Group A
Paranarella watlingi (USNM1096721)
1A
1B1
Narella sp. n A [sensu T&R 2015] (SW3746)
Abyssoprimnoa gemina (USNM1268847)
Narella sp. n B [sensu T&R 2015] (SW3719)
Narella sp. n C [sensu T&R 2015] (SW3807)
Narella macrocalyx (USNM1149566) Group C
Narella hypsocalyx (USNM1180223) Group B
Narella c.f. mosaica (AR121) Group A
Narella arbuscula (USNM1080452) Group C
Narella clavata (AR236) Group A
Narella s.p. n A [sensu T&R 2015] (SW3824)
1B
Candidella helminthophora (N64357)
Parastenella ramosa (USNM1148053)
Parastenella pacifica (N94401)
Parastenella spinosa (G60)
Parastenella c.f. ramosa (SW0208)
Parastenella spinosa (T147)
1B2
Loboprimnoa exotica (N72423)
Primnoa notialis (USNM1278381)
Primnoa notialis (USNM1278382)
Australogorgia aldersladei (USNM1116407)
Primnoa pacifica (USNM1130921)
Primnoa resedaeformis (USNM1024423)
Primnoa notialis (N11292)
Primnoa notialis (N11301)
Primnoa wingi (USNM1115520)
Primnoa s.p. n A [sensu T&R 2015] (SW0960)
Primnoa s.p. n A (SW3518) [sensu T&R 2015]
Primnoa pacifica willeyi (USNM1099637)
Pyrogorgia lemnos (USNM78654)
ML
various, but usually over 5 = 0.96
2
Pachyprimnoa asakoae (N88630)
Perissogorgia vitrea (N9882)
Perissogorgia vitrea (AR202)
Perissogorgia vitrea (AR205)
3
Perissogorgia monile (AR244)
Callogorgia n. sp. (USNM1161750)
Callogorgia gilberti (N76752)
Fanellia medialis (AR210)
Fanellia c.f. tuberculata granulosa (AR220)
Fanellia euthyeia (AR213)
4
Callogorgia sp. (AR201)
Callogorgia sp. (AR119)
Callogorgia sp. (AR223)
1C
Thouarella coronata (SW3809) Group D
Fanellia korema (AR204)
Fanellia korema (AR226)
Character 7: Number of scales in each abaxial body wall row or abaxial face
Parsimony ancestral state reconstruction
variable, but usually over 5
fixed (3 or 4)
fixed (5)
fixed (2)
none
Convexella magelhaenica (FA78)
Heptaprimnoa patagonica (USNM1174977)
Dasystenella acanthina (AR054)
Dasystenella acanthina (PRIM05)
Dasystenella acanthina (C6)
Dasystenella acanthina (N33)
Dasystenella austasensis (AE021)
5A1
2A
Metafannyella eos (N42561)
Plumarella (Faxiella) delicatula (N72100)
Metafannyella ventilabrum (N73567)
Metafannyella polita (N99702)
Aglaoprimnoa stefani (USNM81288)
Metafannyella kuekenthali (I08)
Metafannyella kuekenthali (D3)
Metafannyella kuekenthali (R23)
5A
Primnoella chilensis (H39)
Primnoella chilensis (T124)
Armadillogorgia sp. (FD11)
Armadillogorgia sp. (FD67)
Relative likelihoods for ancestral states discussed in text are shown. For all likelihoods see Additional File 5.
Convexella c.f. magelhaenica (O22)
Convexella sp. (FP65)
Convexella magelhaenica (USNM1232168)
Armadillogorgia cyathella (USNM77148)
Plumarella (Plumarella) pourtalesii (USNM79782)
Plumarella (Verticillata) castellviae (USNM1019337)
Convexella magelhaenica (USNM1113007)
3A
5A2
Primnoella antarctica (AE056)
Primnoella antarctica (N25)
Primnoella antarctica (N26)
Primnoella scotiae (L25)
Primnoella scotiae (B32)
Primnoella scotiae (I25)
Primnoella insularis (USNM1278164)
Primnoella divaricata (PRIM15)
Primnoella c.f. delicatissima (PRIM01)
Primnoella c.f. delicatissima (PRIM02)
Arntzia gracilis (USNM1185335)
Arntzia gracilis (AR002)
Arntzia gracilis (AR058)
Primnoella sp. (USNM1278170)
Ophidiogorgia sp. (USNM1159691)
Ophidiogorgia c.f. kuekenthali (AR131)
Ophidiogorgia kuekenthali (AR099)
Ophidiogorgia c.f. kuekenthali (AR133)
Ophidiogorgia kuekenthali (AR166)
Ophidiogorgia c.f. kuekenthali (AR170)
Digitogorgia kuekenthali (O19)
Primnoeides sertularoides (SW3181)
Primnoeides sertularoides (SW3149)
Primnoeides sertularoides (SW3165)
Primnoeides sertularoides (SW3202)
Primnoeides sp. n. A [sensu T&R 2015] (SW3564)
Primnoeides sertularoides (SW3174)
Primnoeides sp. n. A [sensu T&R 2015] (SW3291)
Primnoeides sp. n. A [sensu T&R 2015] (SW3297)
5
Onogorgia nodosa (USNM82940)
Onogorgia nodosa (USNM1184513)
Onogorgia nodosa (AE010)
Onogorgia nodosa (N29)
Onogorgia nodosa (N28)
Onogorgia nodosa (N30)
Fannyella (Scyphogorgia) abies (N60495)
Fannyella (Fannyella) rossi (AR019)
Fannyella (Fannyella) rossi (AR048)
Fannyella (Fannyella) rossi (AR095)
Fannyella (Fannyella) rossi (AR102)
Fannyella (Fannyella) rossi (AR167)
Fannyella (Fannyella) rossi (N37224)
Fannyella (Cyathogorgia) spinosa (N35534)
Fannyella (Cyathogorgia) spinosa (AR085)
Fannyella (Cyathogorgia) spinosa (AR123)
Fannyella (Cyathogorgia) spinosa (AR173)
Fannyella (Cyathogorgia) spinosa (AR013)
Ainigmaptilon c.f. edisto (N01)
Ainigmaptilon edisto (AR105)
5B1
Callozostron mirabile (N77603)
Callozostron acanthodes (N78759)
Callozostron acanthodes (AR185)
Callozostron pinnatum (N82682)
Plumarella (Plumarella) laxiramosa (USNM1019406)
Plumarella (Plumarella) longispina (USNM1117110)
Plumarella (Dicholaphis) aleutiana (USNM1134746)
Plumarella c.f. delicatissima (N05)
Plumarella (Plumarella) delicatissima (O24)
5B2
Tokoprymno sp. n. [sensu T&R 2015] (PRIM10)
Tokoprymno sp. n [sensu T&R 2015] (PRIM09)
5B
Mirostenella sp. n [sensu T&R 2015] (AP12)
Mirostenella articulata (AC35)
Mirostenella articulata (AE002)
Mirostenella articulata (AE093)
Mirostenella articulata (AE112)
Thouarella c.f. laxa (AR232) Group C
Thouarella laxa (AR194) Group C
Thouarella laxa (AR200) Group C
Thouarella variabilis (AE059) Group A
Thouarella c.f. brucei (T125) Group B
Thouarella c.f. variabilis (AR096) Group A
Thouarella variabilis (AR134) Group A
Thouarella variabilis (AR169) Group A
5B3
Thouarella pendulina (AR135) Group A
Thouarella chilensis (N09) Group A
Thouarella variabilis (AR027) Group A
Thouarella cf variabilis (A06) Group A
Thouarella sp. n. A [sensu T&R 2015] (AC28)
Thouarella variabilis (F6) Group A
Thouarella antarctica (AR018) Group A
Plumarella (Plumarella) diadema (Z051)
Plumarella (Plumarella) diadema (AE068)
Plumarella (Plumarella) diadema (S060)
Plumarella (Plumarella) diadema (B34)
Plumarella (Plumarella) undulata (Z046)
Plumarella (Plumarella) undulata (O28)
Plumarella (Plumarella) undulata (B48)
Plumarella (Plumarella) undulata (S093)
Thouarella viridis (AC23) Group A
Thouarella viridis (AE019) Group A
Thouarella viridis (H13) Group A
Thouarella chilensis (R54) Group A
Thouarella antarctica (AE064) Group A
Thouarella chilensis (AE026) Group A
Thouarella antarctica (S170) Group A
Thouarella crenelata (S125) Group A
Thouarella crenelata (W54) Group A
Thouarella crenelata (AE057) Group A
Thouarella crenelata (AR091) Group A
Thouarella cf antarctica (AR175) Group A
Figure S1g

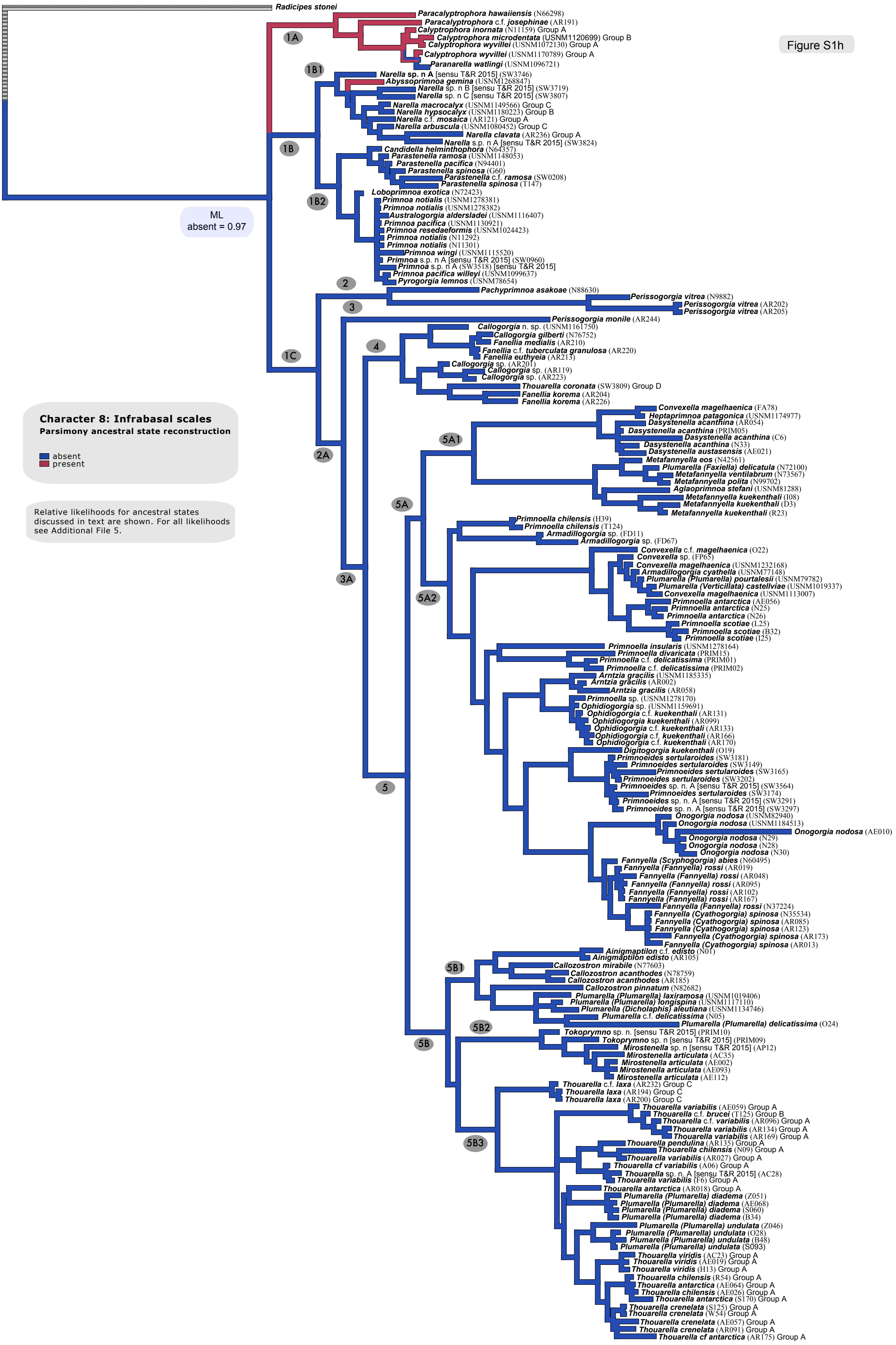

Radicipes stonei
Paracalyptrophora hawaiiensis (N66298)
Paracalyptrophora c.f. josephinae (AR191)
Calyptrophora inornata (N11159) Group A
Calyptrophora microdentata (USNM1120699) Group B
Calyptrophora wyvillei (USNM1072130) Group A
Calyptrophora wyvillei (USNM1170789) Group A
Paranarella watlingi (USNM1096721)
Narella sp. n A [sensu T&R 2015] (SW3746)
Abyssoprimnoa gemina (USNM1268847)
Narella sp. n B [sensu T&R 2015] (SW3719)
Narella sp. n C [sensu T&R 2015] (SW3807)
Narella macrocalyx (USNM1149566) Group C
Narella hypsocalyx (USNM1180223) Group B
Narella c.f. mosaica (AR121) Group A
Narella arbuscula (USNM1080452) Group C
Narella clavata (AR236) Group A
Narella s.p. n A [sensu T&R 2015] (SW3824)
Candidella helminthophora (N64357)
Parastenella ramosa (USNM1148053)
Parastenella pacifica (N94401)
Parastenella spinosa (G60)
Parastenella c.f. ramosa (SW0208)
Parastenella spinosa (T147)
Loboprimnoa exotica (N72423)
Primnoa notialis (USNM1278381)
Primnoa notialis (USNM1278382)
Australogorgia aldersladei (USNM1116407)
Primnoa pacifica (USNM1130921)
Primnoa resedaeformis (USNM1024423)
Primnoa notialis (N11292)
Primnoa notialis (N11301)
Primnoa wingi (USNM1115520)
Primnoa s.p. n A [sensu T&R 2015] (SW0960)
Primnoa s.p. n A (SW3518) [sensu T&R 2015]
Primnoa pacifica willeyi (USNM1099637)
Pyrogorgia lemnos (USNM78654)
Pachyprimnoa asakoae (N88630)
Perissogorgia vitrea (N9882)
Perissogorgia vitrea (AR202)
Perissogorgia vitrea (AR205)
Perissogorgia monile (AR244)
Callogorgia n. sp. (USNM1161750)
Callogorgia gilberti (N76752)
Fanellia medialis (AR210)
Fanellia c.f. tuberculata granulosa (AR220)
Fanellia euthyeia (AR213)
Callogorgia sp. (AR201)
Callogorgia sp. (AR119)
Callogorgia sp. (AR223)
Thouarella coronata (SW3809) Group D
Fanellia korema (AR204)
Fanellia korema (AR226)
Convexella magelhaenica (FA78)
Heptaprimnoa patagonica (USNM1174977)
Dasystenella acanthina (AR054)
Dasystenella acanthina (PRIM05)
Dasystenella acanthina (C6)
Dasystenella acanthina (N33)
Dasystenella austasensis (AE021)
Metafannyella eos (N42561)
Plumarella (Faxiella) delicatula (N72100)
Metafannyella ventilabrum (N73567)
Metafannyella polita (N99702)
Aglaoprimnoa stefani (USNM81288)
Metafannyella kuekenthali (I08)
Metafannyella kuekenthali (D3)
Metafannyella kuekenthali (R23)
Primnoella chilensis (H39)
Primnoella chilensis (T124)
Armadillogorgia sp. (FD11)
Armadillogorgia sp. (FD67)
Convexella c.f. magelhaenica (O22)
Convexella sp. (FP65)
Convexella magelhaenica (USNM1232168)
Armadillogorgia cyathella (USNM77148)
Plumarella (Plumarella) pourtalesii (USNM79782)
Plumarella (Verticillata) castellviae (USNM1019337)
Convexella magelhaenica (USNM1113007)
Primnoella antarctica (AE056)
Primnoella antarctica (N25)
Primnoella antarctica (N26)
Primnoella scotiae (L25)
Primnoella scotiae (B32)
Primnoella scotiae (I25)
Primnoella insularis (USNM1278164)
Primnoella divaricata (PRIM15)
Primnoella c.f. delicatissima (PRIM01)
Primnoella c.f. delicatissima (PRIM02)
Arntzia gracilis (USNM1185335)
Arntzia gracilis (AR002)
Arntzia gracilis (AR058)
Primnoella sp. (USNM1278170)
Ophidiogorgia sp. (USNM1159691)
Ophidiogorgia c.f. kuekenthali (AR131)
Ophidiogorgia kuekenthali (AR099)
Ophidiogorgia c.f. kuekenthali (AR133)
Ophidiogorgia c.f. kuekenthali (AR166)
Ophidiogorgia c.f. kuekenthali (AR170)
Digitogorgia kuekenthali (O19)
Primnoeides sertularoides (SW3181)
Primnoeides sertularoides (SW3149)
Primnoeides sertularoides (SW3165)
Primnoeides sertularoides (SW3202)
Primnoeides sp. n. A [sensu T&R 2015] (SW3564)
Primnoeides sertularoides (SW3174)
Primnoeides sp. n. A [sensu T&R 2015] (SW3291)
Primnoeides sp. n. A [sensu T&R 2015] (SW3297)
Onogorgia nodosa (USNM82940)
Onogorgia nodosa (USNM1184513)
Onogorgia nodosa (AE010)
Onogorgia nodosa (N29)
Onogorgia nodosa (N28)
Onogorgia nodosa (N30)
Fannyella (Scyphogorgia) abies (N60495)
Fannyella (Fannyella) rossi (AR019)
Fannyella (Fannyella) rossi (AR048)
Fannyella (Fannyella) rossi (AR095)
Fannyella (Fannyella) rossi (AR102)
Fannyella (Fannyella) rossi (AR167)
Fannyella (Fannyella) rossi (N37224)
Fannyella (Cyathogorgia) spinosa (N35534)
Fannyella (Cyathogorgia) spinosa (AR085)
Fannyella (Cyathogorgia) spinosa (AR123)
Fannyella (Cyathogorgia) spinosa (AR173)
Fannyella (Cyathogorgia) spinosa (AR013)
Ainigmaptilon c.f. edisto (N01)
Ainigmaptilon edisto (AR105)
Callozostron mirabile (N77603)
Callozostron acanthodes (N78759)
Callozostron acanthodes (AR185)
Callozostron pinnatum (N82682)
Plumarella (Plumarella) laxiramosa (USNM1019406)
Plumarella (Plumarella) longispina (USNM1117110)
Plumarella (Dicholaphis) aleutiana (USNM1134746)
Plumarella c.f. delicatissima (N05)
Plumarella (Plumarella) delicatissima (O24)
Tokoprymno sp. n. [sensu T&R 2015] (PRIM10)
Tokoprymno sp. n [sensu T&R 2015] (PRIM09)
Mirostenella sp. n [sensu T&R 2015] (AP12)
Mirostenella articulata (AC35)
Mirostenella articulata (AE002)
Mirostenella articulata (AE093)
Mirostenella articulata (AE112)
Thouarella c.f. laxa (AR232) Group C
Thouarella laxa (AR194) Group C
Thouarella laxa (AR200) Group C
Thouarella variabilis (AE059) Group A
Thouarella c.f. brucei (T125) Group B
Thouarella c.f. variabilis (AR096) Group A
Thouarella variabilis (AR134) Group A
Thouarella variabilis (AR169) Group A
Thouarella pendulina (AR135) Group A
Thouarella chilensis (N09) Group A
Thouarella variabilis (AR027) Group A
Thouarella cf variabilis (A06) Group A
Thouarella sp. n. A [sensu T&R 2015] (AC28)
Thouarella variabilis (F6) Group A
Thouarella antarctica (AR018) Group A
Plumarella (Plumarella) diadema (Z051)
Plumarella (Plumarella) diadema (AE068)
Plumarella (Plumarella) diadema (S060)
Plumarella (Plumarella) diadema (B34)
Plumarella (Plumarella) undulata (Z046)
Plumarella (Plumarella) undulata (O28)
Plumarella (Plumarella) undulata (B48)
Plumarella (Plumarella) undulata (S093)
Thouarella viridis (AC23) Group A
Thouarella viridis (AE019) Group A
Thouarella viridis (H13) Group A
Thouarella chilensis (R54) Group A
Thouarella antarctica (AE064) Group A
Thouarella chilensis (AE026) Group A
Thouarella antarctica (S170) Group A
Thouarella crenelata (S125) Group A
Thouarella crenelata (W54) Group A
Thouarella crenelata (AE057) Group A
Thouarella crenelata (AR091) Group A
Thouarella cf antarctica (AR175) Group A
1A
1B1
1B
1B2
1C
2
3
2A
4
3A
5A1
5A
5A2
5
5B1
5B2
5B
5B3
ML
absent = 0.97
Character 8: Infrabasal scales
Parsimony ancestral state reconstruction
absent
present
Relative likelihoods for ancestral states discussed in text are shown. For all likelihoods see Additional File 5.

Figure S1h

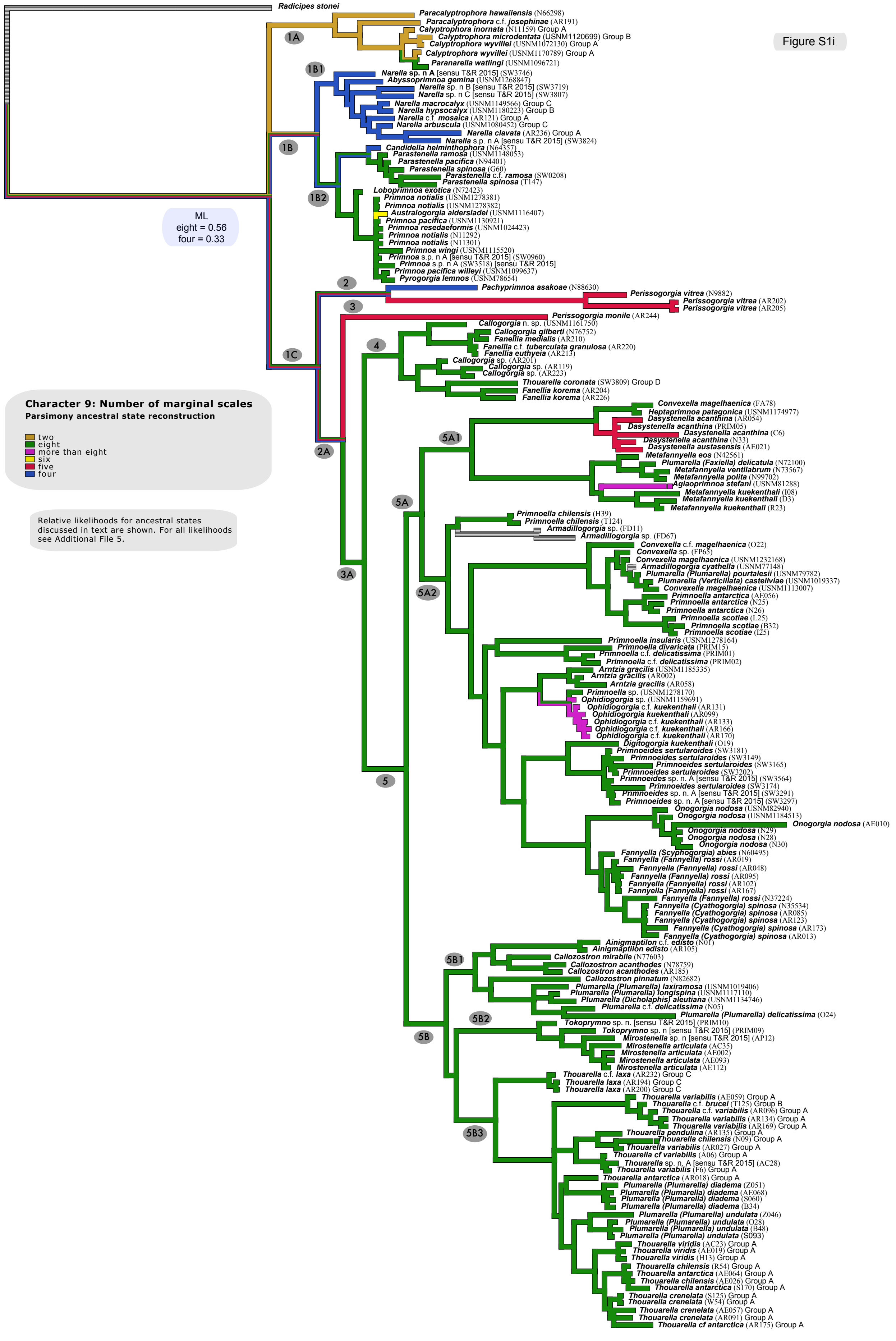

Radicipes stonei
Paracalyptrophora hawaiiensis (N66298)
Paracalyptrophora c.f. josephinae (AR191)
Calyptrophora inornata (N11159) Group A
Calyptrophora microdentata (USNM1120699) Group B
Calyptrophora wyvillei (USNM1072130) Group A
Calyptrophora wyvillei (USNM1170789) Group A
Paranarella watlingi (USNM1096721)
Narella sp. n A [sensu T&R 2015] (SW3746)
Abyssoprimnoa gemina (USNM1268847)
Narella sp. n B [sensu T&R 2015] (SW3719)
Narella sp. n C [sensu T&R 2015] (SW3807)
Narella macrocalyx (USNM1149566) Group C
Narella hypsocalyx (USNM1180223) Group B
Narella c.f. mosaica (AR121) Group A
Narella arbuscula (USNM1080452) Group C
Narella clavata (AR236) Group A
Narella s.p. n A [sensu T&R 2015] (SW3824)
Candidella helminthophora (N64357)
Parastenella ramosa (USNM1148053)
Parastenella pacifica (N94401)
Parastenella spinosa (G60)
Parastenella c.f. ramosa (SW0208)
Parastenella spinosa (T147)
Loboprimnoa exotica (N72423)
Primnoa notialis (USNM1278381)
Primnoa notialis (USNM1278382)
Australogorgia aldersladei (USNM1116407)
Primnoa pacifica (USNM1130921)
Primnoa resedaeformis (USNM1024423)
Primnoa notialis (N11292)
Primnoa notialis (N11301)
Primnoa wingi (USNM1115520)
Primnoa s.p. n A [sensu T&R 2015] (SW0960)
Primnoa s.p. n A (SW3518) [sensu T&R 2015]
Primnoa pacifica willeyi (USNM1099637)
Pyrogorgia lemnos (USNM78654)
Pachyprimnoa asakoae (N88630)
Perissogorgia vitrea (N9882)
Perissogorgia vitrea (AR202)
Perissogorgia vitrea (AR205)
Perissogorgia monile (AR244)
Callogorgia n. sp. (USNM1161750)
Callogorgia gilberti (N76752)
Fanellia medialis (AR210)
Fanellia c.f. tuberculata granulosa (AR220)
Fanellia euthyeia (AR213)
Callogorgia sp. (AR201)
Callogorgia sp. (AR119)
Callogorgia sp. (AR223)
Thouarella coronata (SW3809) Group D
Fanellia korema (AR204)
Fanellia korema (AR226)
Convexella magelhaenica (FA78)
Heptaprimnoa patagonica (USNM1174977)
Dasystenella acanthina (AR054)
Dasystenella acanthina (PRIM05)
Dasystenella acanthina (C6)
Dasystenella acanthina (N33)
Dasystenella austasensis (AE021)
Metafannyella eos (N42561)
Plumarella (Faxiella) delicatula (N72100)
Metafannyella ventilabrum (N73567)
Metafannyella polita (N99702)
Aglaoprimnoa stefani (USNM81288)
Metafannyella kuekenthali (I08)
Metafannyella kuekenthali (D3)
Metafannyella kuekenthali (R23)
Primnoella chilensis (H39)
Primnoella chilensis (T124)
Armadillogorgia sp. (FD11)
Armadillogorgia sp. (FD67)
Convexella c.f. magelhaenica (O22)
Convexella sp. (FP65)
Convexella magelhaenica (USNM1232168)
Armadillogorgia cyathella (USNM77148)
Plumarella (Plumarella) pourtalesii (USNM79782)
Plumarella (Verticillata) castellviae (USNM1019337)
Convexella magelhaenica (USNM1113007)
Primnoella antarctica (AE056)
Primnoella antarctica (N25)
Primnoella antarctica (N26)
Primnoella scotiae (L25)
Primnoella scotiae (B32)
Primnoella scotiae (I25)
Primnoella insularis (USNM1278164)
Primnoella divaricata (PRIM15)
Primnoella c.f. delicatissima (PRIM01)
Primnoella c.f. delicatissima (PRIM02)
Arntzia gracilis (USNM1185335)
Arntzia gracilis (AR002)
Arntzia gracilis (AR058)
Primnoella sp. (USNM1278170)
Ophidiogorgia sp. (USNM1159691)
Ophidiogorgia c.f. kuekenthali (AR131)
Ophidiogorgia kuekenthali (AR099)
Ophidiogorgia c.f. kuekenthali (AR133)
Ophidiogorgia c.f. kuekenthali (AR166)
Ophidiogorgia c.f. kuekenthali (AR170)
Digitogorgia kuekenthali (O19)
Primnoeides sertularoides (SW3181)
Primnoeides sertularoides (SW3149)
Primnoeides sertularoides (SW3165)
Primnoeides sertularoides (SW3202)
Primnoeides sp. n. A [sensu T&R 2015] (SW3564)
Primnoeides sertularoides (SW3174)
Primnoeides sp. n. A [sensu T&R 2015] (SW3291)
Primnoeides sp. n. A [sensu T&R 2015] (SW3297)
Onogorgia nodosa (USNM82940)
Onogorgia nodosa (USNM1184513)
Onogorgia nodosa (AE010)
Onogorgia nodosa (N29)
Onogorgia nodosa (N28)
Onogorgia nodosa (N30)
Fannyella (Scyphogorgia) abies (N60495)
Fannyella (Fannyella) rossi (AR019)
Fannyella (Fannyella) rossi (AR048)
Fannyella (Fannyella) rossi (AR095)
Fannyella (Fannyella) rossi (AR102)
Fannyella (Fannyella) rossi (AR167)
Fannyella (Fannyella) rossi (N37224)
Fannyella (Cyathogorgia) spinosa (N35534)
Fannyella (Cyathogorgia) spinosa (AR085)
Fannyella (Cyathogorgia) spinosa (AR123)
Fannyella (Cyathogorgia) spinosa (AR173)
Fannyella (Cyathogorgia) spinosa (AR013)
Ainigmaptilon c.f. edisto (N01)
Ainigmaptilon edisto (AR105)
Callozostron mirabile (N77603)
Callozostron acanthodes (N78759)
Callozostron acanthodes (AR185)
Callozostron pinnatum (N82682)
Plumarella (Plumarella) laxiramosa (USNM1019406)
Plumarella (Plumarella) longispina (USNM1117110)
Plumarella (Dicholaphis) aleutiana (USNM1134746)
Plumarella c.f. delicatissima (N05)
Plumarella (Plumarella) delicatissima (O24)
Tokoprymno sp. n. [sensu T&R 2015] (PRIM10)
Tokoprymno sp. n [sensu T&R 2015] (PRIM09)
Mirostenella sp. n [sensu T&R 2015] (AP12)
Mirostenella articulata (AC35)
Mirostenella articulata (AE002)
Mirostenella articulata (AE093)
Mirostenella articulata (AE112)
Thouarella c.f. laxa (AR232) Group C
Thouarella laxa (AR194) Group C
Thouarella laxa (AR200) Group C
Thouarella variabilis (AE059) Group A
Thouarella c.f. brucei (T125) Group B
Thouarella c.f. variabilis (AR096) Group A
Thouarella variabilis (AR134) Group A
Thouarella variabilis (AR169) Group A
Thouarella pendulina (AR135) Group A
Thouarella chilensis (N09) Group A
Thouarella variabilis (AR027) Group A
Thouarella cf variabilis (A06) Group A
Thouarella sp. n. A [sensu T&R 2015] (AC28)
Thouarella variabilis (F6) Group A
Thouarella antarctica (AR018) Group A
Plumarella (Plumarella) diadema (Z051)
Plumarella (Plumarella) diadema (AE068)
Plumarella (Plumarella) diadema (S060)
Plumarella (Plumarella) diadema (B34)
Plumarella (Plumarella) undulata (Z046)
Plumarella (Plumarella) undulata (O28)
Plumarella (Plumarella) undulata (B48)
Plumarella (Plumarella) undulata (S093)
Thouarella viridis (AC23) Group A
Thouarella viridis (AE019) Group A
Thouarella viridis (H13) Group A
Thouarella chilensis (R54) Group A
Thouarella antarctica (AE064) Group A
Thouarella chilensis (AE026) Group A
Thouarella antarctica (S170) Group A
Thouarella crenelata (S125) Group A
Thouarella crenelata (W54) Group A
Thouarella crenelata (AE057) Group A
Thouarella crenelata (AR091) Group A
Thouarella cf antarctica (AR175) Group A
1A
1B1
1B
1B2
2
3
1C
4
2A
5A1
5A
3A
5A2
5
5B1
5B2
5B
5B3
ML
eight = 0.56
four = 0.33
Character 9: Number of marginal scales
Parsimony ancestral state reconstruction
two
eight
more than eight
six
five
four
Relative likelihoods for ancestral states discussed in text are shown. For all likelihoods see Additional File 5.

Figure S1i
